# Community Analysis-based Screening of Plant Growth-promoting Bacteria for Sugar Beet

**DOI:** 10.1264/jsme2.ME20137

**Published:** 2021-04-27

**Authors:** Kazuyuki Okazaki, Hirohito Tsurumaru, Megumi Hashimoto, Hiroyuki Takahashi, Takashi Okubo, Takuji Ohwada, Kiwamu Minamisawa, Seishi Ikeda

**Affiliations:** 1 Memuro Research Station, Hokkaido Agricultural Research Center, National Agriculture and Food Research Organization, 9–4 Shinsei-minami, Memuro, Kasai-gun, Hokkaido 082–0081, Japan; 2 Graduate School of Life Science, Tohoku University, 2–1–1 Katahira, Aoba-ku, Sendai, Miyagi 980–8577, Japan; 3 Department of Agricultural and Life Sciences, Obihiro University of Agriculture and Veterinary Medicine, Obihiro, Hokkaido 080–8555, Japan

**Keywords:** 16S rRNA gene, biofertilizer, community analysis, plant growth-promoting bacteria, sugar beet

## Abstract

Clone libraries of bacterial 16S rRNA genes (a total of 1,980 clones) were constructed from the leaf blades, petioles, taproots, and lateral roots of sugar beet (*Beta vulgaris* L.) grown under different fertilization conditions. A principal coordinate analysis revealed that the structures of bacterial communities in above- and underground tissues were largely separated by PC1 (44.5%). The bacterial communities of above-ground tissues (leaf blades and petioles) were more tightly clustered regardless of differences in the tissue types and fertilization conditions than those of below-ground tissues (taproots and lateral roots). The bacterial communities of below-ground tissues were largely separated by PC2 (26.0%). To survey plant growth-promoting bacteria (PGPBs), isolate collections (a total of 665 isolates) were constructed from the lateral roots. As candidate PGPBs, 44 isolates were selected via clustering analyses with the combined 16S rRNA gene sequence data of clone libraries and isolate collections. The results of inoculation tests using sugar beet seedlings showed that eight isolates exhibited growth-promoting effects on the seedlings. Among them, seven isolates belonging to seven genera (*Asticcacaulis*, *Mesorhizobium*, *Nocardioides*, *Sphingobium*, *Sphingomonas*, *Sphingopyxis*, and *Polaromonas*) were newly identified as PGPBs for sugar beet at the genus level, and two isolates belonging to two genera (*Asticcacaulis* and *Polaromonas*) were revealed to exert growth-promoting effects on the plant at the genus level for the first time. These results suggest that a community analysis-based selection strategy will facilitate the isolation of novel PGPBs and extend the potential for the development of novel biofertilizers.

Approximately 20% of the world’s sucrose production is derived from sugar beet (*Beta vulgaris* L.), the most important crop in temperate regions for sugar production (Godshall, 2012). Although the initial growth of sugar beet seedlings is often inhibited by environmental stress, such as nutrient deficiency and frost damage, sugar beet grows well under harsh environmental conditions once initial growth is established (Steingrobe, 2001; 2005). Sugar beet has recently been attracting attention as a source of bioenergy (Koga, 2008) because of its higher biomass production than other temperate crops (de Vries *et al.*, 2010). However, the mechanisms underlying the high productivity and stress tolerance of sugar beet have not yet been elucidated in detail. One possible explanation for these features may be the colonization of plant growth-promoting bacteria (PGPBs), which confer stress tolerance and growth-promoting effects in the seedling stage (Steingrobe, 2005; Toyota and Watanabe, 2013). To date, a number of bacterial species have been reported as PGPBs for sugar beet, including *Acinetobacter* (Shi *et al.*, 2009, 2010), *Bacillus* (Çakmakçi *et al.*, 1999, 2001; Shi *et al.*, 2009, 2010), *Burkholderia* (Çakmakçi *et al.*, 2001), *Chryseobacterium* (Shi *et al.*, 2009, 2010), *Pseudomonas* (Kloepper *et al.*, 1980; Dunne *et al.*, 1998; Çakmakçi *et al.*, 2001, 2006), *Paenibacillus* (Çakmakçi *et al.*, 2006), *Rhodobacter* (Çakmakçi *et al.*, 2006), and *Stenotrophomonas* spp. (Dunne *et al.*, 1998). However, the phylogenetic diversity of these PGPBs is limited to a certain range of taxonomic groups when considering the entire phylogenetic diversity of sugar beet-associated bacteria, as revealed in our previous studies (Okazaki *et al.*, 2014; Tsurumaru *et al.*, 2015). Surveys of PGPBs have frequently been conducted on the basis of plant‍ ‍growth-promoting traits (PGPTs), including 1-aminocyclopropane-1-carboxylic acid deaminase production, indole acetic acid (IAA) production, N_2_ fixation, phosphate solubilization, pyrroloquinoline quinone production, siderophore production, and plant disease suppression (Dunne *et al.*, 1998; Çakmakçi *et al.*, 2001; Ahmad *et al.*, 2008; Shi *et al.*, 2009; Kumar *et al.*, 2012; Tani *et al.*, 2012; Bal *et al.*, 2013; Gupta *et al.*, 2014). However, assays for these traits are both time- and labor-intensive, which limits large-scale surveys on PGPBs. Recent studies revealed that a single PGPT is not a fully reliable marker for selecting PGPBs; multiple PGPTs are considered to contribute to the beneficial effects of a PGPB (Ahmad *et al.*, 2008; Kumar *et al.*, 2012; Quecine *et al.*, 2012). In addition, bacterial isolates selected using known PGPTs often fail to produce the desired growth-promoting effects when they are inoculated onto plants even under experimental conditions, such as a growth chamber or greenhouse. Therefore, screening using known PGPTs is not currently considered to be an efficient strategy for identifying PGPBs. One possible explanation for this failure is the insufficient consideration of the colonization ability of PGPBs on plant tissue (Lugtenberg and Kamilova, 2009; Compant *et al.*, 2010).

It is conceivable that the colonization ability of a plant-associated microorganism is reflected in its abundance in the tissue of a plant species. Therefore, a community analysis-based screening of PGPBs may be an ecologically reasonable and powerful strategy for identifying and selecting novel PGPBs that are highly compatible with a plant (tissue). The diversity of plant-associated bacteria is also markedly affected by fertilization conditions in several major crops (Ikeda *et al.*, 2014; Unno *et al.*, 2015; Masuda *et al.*, 2016). Stable colonization on and in plant tissues under diverse environmental conditions is regarded as a favorite characteristic of PGPBs for their use in agricultural practice under field conditions. A metagenomic analysis of the phylogenetic diversity and functionality of taproot-associated bacterial community in sugar beet (Tsurumaru *et al.*, 2015) revealed the dominance of *Alphaproteobacteria* in taproot tissue, which is consistent with the findings of Shi *et al.* (2014), revealing the potential importance of the functionality of this bacterial group for high biomass production by sugar beet. Collectively, these findings suggest that *Alphaproteobacteria* is a promising candidate group for screening PGPBs for sugar beet.

To obtain a more detailed understanding of the role of plant-associated bacteria in the growth of sugar beet, the present study aimed to (i) reveal dominant bacterial groups in the above- and underground tissues of sugar beet under different fertilization conditions via a clone library analysis, (ii) build bacterial isolate collections as a resource for surveying PGPBs, and (iii) conduct a large-scale screening of PGPBs for sugar beet by employing the combined data of 16S rRNA gene sequences derived from clone libraries and isolate collections. As a result, a subset of novel PGPBs were efficiently selected and identified for sugar beet at the genus and species levels, indicating that a community analysis-based screening strategy is a powerful tool for surveying and selecting novel PGPBs for practical agricultural use.

## Materials and Methods

### Plant materials and sampling

Seeds of the sugar beet cultivar “Amahomare” were sown in pots (paper pot no. 1; Nippon Beet Sugar Manufacturing) under greenhouse conditions on March 16, 2010 and grown for 41 days. They were planted in a plot with standard fertilization (NPK plot), only P and K fertilization (PK plot, no N fertilization), or only K fertilization (K plot, no N or P fertilization). All plots were 31.2 m^2^ in size, and planting was performed on April 26, 2010 in a long-term experimental field in Japan (42°89′20″N, 143°07′70″E, 94‍ ‍m a.s.l.) that had been maintained under the rotation of upland crops with potato, maize, sugar beet, or soybean grown during the summer and no cultivation during the winter since 1994 at the Memuro Research Station of the Hokkaido Agricultural Research Center (Memuro, Hokkaido, Japan). Ammonium sulfate (150 kg of N hectare^–1^ for the NPK plot), calcium superphosphate (250 kg of P_2_O_5_ hectare^–1^ for the NPK and PK plots), and potassium sulfate (160 kg of K_2_O kg hectare^–1^ for the NPK, PK, and K plots) were applied as basal fertilizers. On July 12, 2010, based on visual inspections, nine healthy plants were randomly sampled from the NPK, PK, and K plots. They were carefully washed with tap water to remove loosely adhering soil and organic debris, rinsed with sterilized water, and individually separated into taproots, lateral roots, leaf blades, and petioles. Lateral roots on a taproot were collected using forceps. These tissues were stored at –30°C until used for the construction of a clone library or bacterial isolate collection. Soil samples from the NPK, PK, and K plots were also taken from between plants using an auger (between 5 and 15‍ ‍cm in depth) at the time of sampling, and the chemical characteristics of these soils were elucidated by the Tokachi Nokyoren Agricultural Research Institute (Obihiro, Hokkaido, Japan). The analysis of each chemical characteristic was conducted using the following methods: pH (H_2_O); pH meter, P_2_O_5_; Truog’s method, K_2_O, MgO, CaO, and CEC; Schollenberger’s method, total nitrogen; dry combustion method, NO_3_-N; hydrazine reduction method, NH_4_-N; indophenol method, Phosphate absorption coefficient; SPAD simple method, according to Nakatsu *et al.* (2012).

### Construction of 16S rRNA gene clone libraries from sugar beet-associated bacteria

The 16S rRNA gene clone libraries of sugar beet-associated bacteria were constructed using the leaf blades, petioles, taproots, and lateral roots of sugar beet grown in the NPK, PK, and K plots. Bacterial cells were individually extracted from the leaf blades, petioles, taproots, and lateral roots of nine plants, as previously reported (Ikeda *et al.*, 2009). Briefly, 25‍ ‍g of leaf blades or petioles were collected from a plant and homogenized in a blender with 250‍ ‍mL of bacterial cell extraction buffer. Taproots were cut into several pieces, and 100‍ ‍g of tissue was homogenized in a blender with 500‍ ‍mL of bacterial cell extraction buffer. Lateral roots (approximately 1 g) were ground in liquid nitrogen with a mortar and pestle and homogenized in a blender with 250‍ ‍mL of bacterial cell extraction buffer. The metagenomic DNA of extracted bacterial cells was prepared according to the protocol of Ikeda *et al.* (2004). DNA samples were used as the template for the PCR amplification of the 16S rRNA gene. PCR amplification was performed using the universal primers 27F (5′-AGAGTTTGATCMTGGCTCAG-3′) and 1525R (5′-AAGGAGGTGWTCCARCC-3′), as previously reported (Someya *et al.*, 2013). After electrophoresis of the PCR products on a 1% agarose gel, amplicons of the expected size (approximately 1,500 bp) were purified using NucleoSpin Extract II (Macherey-Nagel). Equal amounts of amplicons derived from each of the nine plants were combined for each tissue and then cloned using the pGEM-T Easy TA cloning vector (Promega). A partial sequence of the 16S rRNA gene (corresponding to bases 109–665 of the gene in *Escherichia coli*) for each clone of the library was elucidated using the 27F primer by Takara Bio.

### Construction of bacterial isolate collections of lateral root-associated bacteria

Lateral root-associated bacteria were isolated from the lateral roots of sugar beet grown in the PK or K plots based on the presumption of the proliferation of beneficial bacteria for plant growth as a compensatory effect under nutrient-limited conditions. Lateral roots derived from three plants grown in a plot (1‍ ‍g from each plant, approximately 3‍ ‍g in total) were homogenized in a mortar and pestle with 30‍ ‍mL of 67‍ ‍mM phosphate buffer (pH 7.0). The homogenate was filtered through a layer of Miracloth (Calbiochem), and an aliquot (100‍ ‍μL) of the filtrate was spread onto a R2A (Becton, Dickinson, and Company) or tryptic soy agar (TSA; Becton, Dickinson, and Company) plate containing cycloheximide (50‍ ‍mg mL^–1^; Wako Pure Chemical Industries) as the antifungal agent. After 1‍ ‍week of cultivation at 24°C in the dark, colonies were randomly selected and subjected to single colony isolation twice. These isolates were suspended into R2A or tryptic soy broth liquid medium containing 15% glycerol and stored at –80°C until later use for DNA extraction and inoculation tests.

The sequencing of 16S rRNA genes for isolate collections was also conducted. Isolates were cultivated on R2A or TSA plates, and a portion of each colony was used for genomic DNA extraction as previously described by Okazaki *et al.* (2014). In addition, a partial sequence of the 16S rRNA gene for each isolate of the collections was elucidated using the 27F primer as previously described for the sequencing of clone libraries. Nearly the full length of the 16S rRNA gene sequence was elucidated for growth-promoting or growth-inhibiting bacteria using the r1L (5′-GTATTACCGCGGCTGCTGG-3′), 926f (5′-AAACTCAAAGGAATTGACGG-3′), and f2L (5′-CCAGCAGCCGCGGTAATAG-3′) primers by Takara Bio.

### Clustering analysis of 16S rRNA gene sequences

Clustering analyses of 16S rRNA gene sequences were performed with the combined data of clone libraries and isolate collections. The sequence orientation and presence of non-16S rRNA gene sequences in the libraries were examined using OrientationChecker (Ashelford *et al.*, 2006). Chimeric sequences in the libraries were removed using MALLARD (Ashelford *et al.*, 2006). Any sequence identified at the 99.9% threshold was discarded as a chimera. The remaining sequences were aligned using CLUSTAL W (Thompson *et al.*, 1994). Based on the alignment, a distance matrix was constructed using the DNADIST program from PHYLIP ver. 3.66 (http://evolution.genetics.washington.edu/phylip.html) with the default parameters. The resulting matrices were used as the input for the Mothur program (Schloss *et al.*, 2009) to create operational taxonomic units (OTUs) with a threshold value of 97% sequence identity and calculate the diversity indices of clone libraries and isolate collections. Library coverage was calculated as described by Kemp and Aller (2004).

### Principal coordinate analysis (PCoA) of 16S rRNA gene clone libraries

The UniFrac program (Lozupone *et al.*, 2006) was applied to examine the similarities in community structures between clone libraries. A tree file generated by CLUSTAL W and an environment file that links a file to the library were used as the input for UniFrac to conduct PCoA with the abundance-weighted option.

### Phylogenetic analysis of 16S rRNA gene sequences

The phylogenetic compositions (the relative abundance of taxa) in clone libraries and isolate collections were analyzed using the Classifier program in the Ribosomal Database Project (RDP; Wang *et al.*, 2007) with a confidence threshold of 80%. Statistical comparisons among clone libraries were conducted using the Library Compare program in RDP (Cole *et al.*, 2014).

### Selection of sugar beet growth-promoting bacteria

To select sugar beet growth-promoting bacteria, OTUs in isolate collections were selected based on their phylogenetic novelty (less than 97% identity with the closest known species), tissue specificity (restricted presence in taproots or lateral roots), persistence among libraries or collections (stable presence regardless of fertilizer application conditions), or high abundance in a tissue (more than 1% relative abundance in a clone library or isolate collection) in taproots or lateral roots. In addition, OTUs exhibiting high identity to the 16S rRNA gene sequences of known PGPBs reported in previous studies were selected. From a practical viewpoint, OTUs displaying high identity to a plant, animal, or human pathogen were eliminated in the selection process of the present study.

One isolate was selected from each of the OTUs matching the criteria described above as an inoculum and cultivated on an R2A or TSA plate at 24°C for 3 days in the dark, and colonies were suspended in sterilized water. The bacterial cell suspension was washed and adjusted to an optical density at 660‍ ‍nm of 0.1 with sterilized water as an inoculant.

Sugar beet seedlings were prepared as follows. Sugar beet seeds (cultivar “Rycka”) were sterilized via soaking in 70% ethanol for 1‍ ‍min followed by 1% sodium hypochlorite (containing 0.01% Tween 20) for 15‍ ‍min. After rinsing with sterilized water, surface-sterilized seeds were covered with wet filter paper. They were placed in a sterile Petri dish and germinated at 25°C for 1 day in the dark. Commercial soil (Pot-ace N; Katakura & Co-op Agri Corporation, 200‍ ‍mg N L^–1^, 800‍ ‍mg P L^–1^, 200‍ ‍mg K L^–1^, 60‍ ‍mg Mg L^–1^) for nursing seedlings was sterilized via autoclaving at 121°C for 5‍ ‍min, and 80‍ ‍mL of soil was added to a pot (41×41×43.5‍ ‍mm^3^; Cell box, Meiwa). Two germinated seeds were planted in a pot and covered with 20‍ ‍mL of soil. One milliliter of the bacterial inoculant was applied to a pot. Control seeds were inoculated with sterilized water. Seedlings were then grown in a plant growth chamber (16 h of light at 25°C and 8 h of darkness at 20°C) (NK system E5ZS-34; Nippon Medical & Chemical Instruments), and distilled water was supplied as needed to maintain the moisture content. After 1‍ ‍week of cultivation, seedlings were thinned to one plant per pot. After 4‍ ‍weeks of cultivation, a whole seedling was sampled and separated into shoots and roots. These tissues were air-dried at 80°C for 3 days and dry matter weight was measured. Twelve seedlings in a tray were used in an inoculation test with each isolate, and this test was repeated three or four times at different dates to ensure the reproducibility of PGP effects. In order to correct data variations among repeated tests at different dates, data for PGP effects were evaluated by Welch’s *t*-tests (two-tailed) using the ratio data of dry weight relative to a control.

### Phylogenetic tree analysis

In the phylogenetic tree analysis, sequences were aligned using the CLUSTAL W program. The neighbor-joining method was used to build the trees (Saitou and Nei, 1987). The PHYLIP format tree output was applied using the bootstrapping procedure with 1,000 replicates (Felsenstein, 1985). Trees were constructed with TreeView software (Page, 1996).

### Statistical analysis

Welch’s *t*-tests (two-tailed) were performed using JMP software version 12 (SAS Institute). A *P* value <0.05 was considered to be significant.

### Accession numbers of nucleotide sequences

Nucleotide sequences were deposited into the DDBJ/EMBL/GenBank database. The sequence data for clone libraries and isolate collections were deposited under the accession nos. LC038237–LC040216 and LC040217–LC040864, respectively (Table S1). The nearly full length of the 16S rRNA gene sequences (approximately 1,400 bp) for sugar beet growth-promoting and growth-inhibiting bacteria were deposited under the accession nos. LC040865–LC040881 and LC602158–LC602165.

## Results and Discussion

### Clone library analyses of sugar beet-associated bacteria under different fertilization conditions

**Fig. 1.**
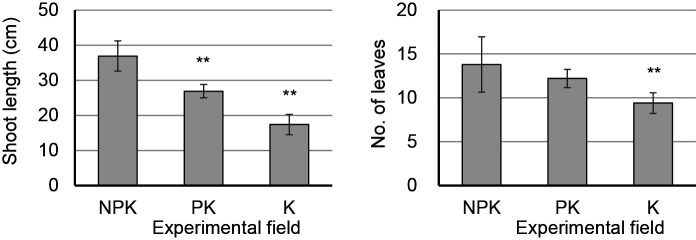
Shoot length and number of leaves on sugar beet plants at the time of sampling. NPK, PK, and K denote plots with standard fertilization, only P and K fertilization (no N fertilization), and only K fertilization (no fertilization with N or P), respectively. Each value indicates the mean±standard deviation of 10 individual plants. Double asterisks indicate a significant difference from NPK by Welch’s *t*-test (two-tailed) at *P*<0.01.

The effects of fertilization conditions on the chemical characteristics of soil in the NPK, PK, and K plots are summarized in Table S2. Although a statistical analysis was not performed due to the lack of replications at the plot level in field experiments, the following changes were generally observed. N levels in soils were similar among the plots. However, the shoot length and number of leaves in the PK and K plots was markedly shorter and smaller, respectively, than those in the NPK plot (Fig. 1).

**Fig. 2.**
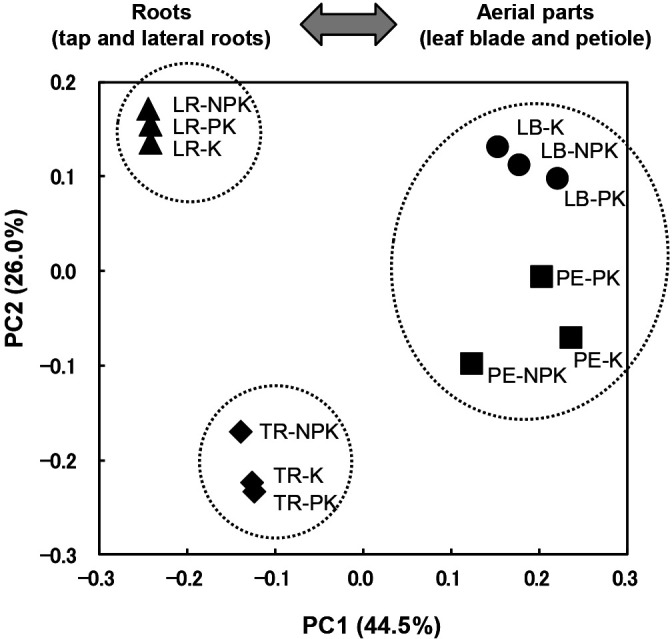
Principal coordinate analysis (PCoA) of 16S rRNA gene sequences of sugar beet-associated bacteria. The library name is indicated on the right side of each symbol. Circles, squares, diamonds, and triangles denote leaf blade (LB)-, petiole (PE)-, taproot (TR)-, and lateral root (LR)-derived libraries, respectively. NPK, PK, and K denote plots with standard fertilization, only P and K fertilization (no N fertilization), and only K fertilization (no fertilization with N or P), respectively.

Library coverages were lower in the libraries for below-ground tissues (61.5–76.4%) than in those for above-ground tissues (88.3–93.3%; Table 1). As expected, the Chao1, ACE, and Shannon indices were higher in the libraries for below-ground tissues than in those for above-ground tissues (Table 1). The numbers of OTUs and the Shannon and Simpson indices were similar between leaf blades and petioles under the same fertilization conditions. Although these diversity indices for leaf blades and petioles were lower in response to the degree of deterioration in fertilization conditions in the PK and K plots than in the NPK plot, the same indices for taproots and lateral roots were both stable under all field conditions. These results suggest that the bacterial diversity of above-ground tissues is more sensitive to fertilization management than that of below-ground tissues. PCoA revealed that the diversity of sugar beet-associated bacteria was mainly clustered into three groups (above-ground tissues, taproots, and lateral roots; Fig. 2). The size of these clusters also suggested that the bacterial diversity of above-ground tissues was more sensitive to fertilization management than that of below-ground tissues in terms of the phylogenetic composition. Fluctuations in the relative abundance of many taxa strongly depended on fertilization management (Tables 2 and 3). As a result of the deterioration of plant nutrition in the PK and K plots, bacterial diversity in the above-ground tissues decreased. The relative abundance and number of OTUs belonging to *Firmicutes* decreased in the PK and K plots (Tables 2 and S3). In isolate collections, *Firmicutes* bacteria were rarely isolated on R2A medium. A high nutritional condition medium may be preferred for the efficient isolation of this bacterial group, or some pretreatment, such as heat shock, to break their dormancy may be required under nutrient-deficient condition. These results most likely reflect the differences in nutrient conditions for bacterial communities between above- and below-ground plant tissues. Therefore, above-ground plant tissue-associated bacteria almost totally depended on most of their nutrients through plant metabolism, while below-ground tissue-associated bacteria, such as some bacterial groups living on roots, depended on their nutrients through not only plant metabolism, but also soil.

Clusters of the bacterial communities of above-ground tissues were separated from those of below-ground tissues along PC1 (44.5%), whereas those of taproots and lateral roots were separated more clearly along PC2 (26.0%) than those for leaf blades and petioles, indicating that taproots and lateral roots harbor unique and distinct bacterial diversity.

Phylogenetic composition analyses revealed that *Proteobacteria*, particularly *Alpha-* and *Gammaproteobacteria*, largely dominated the entire phytosphere of sugar beet (Table 2). In *Alphaproteobacteria*, *Rhizobiales* was exclusively found in the leaf blades, petioles, and taproots, followed by *Sphingomonadales* with less abundance. Two genera, namely, *Methylobacterium* and *Phyllobacterium*, were mainly responsible for the high abundance of *Rhizobiales* in above-ground tissues (Table 3). The high abundance of *Methylobacterium* in above-ground tissues (approximately 15–46%) was also observed in other plant species (Delmotte *et al.*, 2009; Someya *et al.*, 2013; Okubo *et al.*, 2014; Minami *et al.*, 2016; Hara *et al.*, 2019). *Devosia*, *Mesorhizobium*, and unclassified *Bradyrhizobiaceae* bacteria were uniquely found in below-ground tissues. Including these genera, the dominance of *Rhizobiales* in taproots (28–39%; Table 2) was demonstrated in our previous metagenome analysis of taproots (Tsurumaru *et al.*, 2015). At the genus level, *Rhizobium* was a common taxonomic group throughout the entire phytosphere of sugar beet, as previously reported for other plant species, such as *Arabidopsis* (Bodenhausen *et al.*, 2013) and potato (Someya *et al.*, 2013; Unno *et al.*, 2015). The high persistency of this genus in various tissues of diverse plant species suggests an unknown ecological role for *Rhizobium* in the phytosphere.

In *Sphingomonadales*, *Sphingomonas* and *Novosphingobium* were dominantly present in sugar beet tissues (Table 3). *Sphingomonas* was mainly found in above-ground tissues. In contrast, *Novosphingobium* was exclusively present in below-ground tissues, particularly in taproots as the most dominant genus. The distribution patterns of these two genera in the sugar beet phytosphere suggested an equivalent ecological role for these genera in the above- and below-ground tissues of sugar beet. The presence of *Novosphingobium* in taproots was reported in our previous metagenome analysis (Tsurumaru *et al.*, 2015); however, the relative abundance of *Novosphingobium* was small (approximately 3%) and the most dominant taxon in taproots was *Mesorhizobium* (14%). These differences between previous findings and the present results may be attributed to the lack of sufficient genomic data for the genus *Novosphingobium*. Alternately, the abundance of dominant taxa may be markedly affected by differences in the methodologies employed, climate conditions, growth stages (a single time point for sampling), and cultivars of sugar beet.

*Gammaproteobacteria* in sugar beet tissues mainly consisted of three taxonomic groups (*Enterobacteriaceae*, *Xanthomonadaceae*, and *Pseudomonadaceae*). *Enterobacteriaceae* and *Xanthomonadaceae* were exclusively found in above-ground tissues and lateral roots, respectively, whereas *Pseudomonadaceae* was detected in all tissues as a dominant genus (Table 2). *Verrucomicrobia* and *Planctomycetes* were mainly observed in below-ground tissues under all fertilization conditions. *Acidobacteria* and *Bacteroidetes* were only detected in lateral roots. The high abundance of *Niastella* in lateral roots is one of the unique characteristics of the sugar beet phytosphere (Table 3). *Niastella* was recently reported to be more abundant in sugarcane roots than in bulk soils (Yeoh *et al.*, 2016). *Niastella* sp. may be aggressively attracted to the rhizospheres of high sugar-accumulating crops.

### Isolate collections of lateral root-associated bacteria

Bacteria were isolated from the lateral roots of sugar beet grown in the PK or K plot using R2A or TSA medium, and four isolate collections were constructed with 665 isolates (Table 1). Alpha diversity indices for the isolate collections revealed that all indices were higher in the isolate collections derived from R2A medium (LR-PK-R and LR-K-R) than in those derived from TSA medium (LR-PK-T and LR-K-T). Marked differences were observed between the LR-K-R and LR-K-T isolate collections. These results suggest that R2A medium is more suitable for isolating phylogenetically diverse bacteria than TSA medium, as reported in our previous studies (Okubo *et al.*, 2009; Okazaki *et al.*, 2014). For example, *Mesorhizobium*, *Neorhizobium*, *Nocardioides*, *Polaromonas*, and *Sphingomonas* were exclusively isolated with R2A medium, but not with TSA medium, whereas *Pantoea* was isolated with TSA medium only (Table 3). Low concentrations of carbon sources or NaCl in R2A medium may contribute to enhancing the propagation of oligotrophic bacteria. Nutrient-rich media, such as TSA and NA media, most of which were developed in medical science, appear to preferentially enhance the propagation of copiotrophic bacteria, such as *Pantoea*.

The results of the phylogenetic composition analysis (Table 3) suggested that the greatest difference between clone libraries and isolate collections was the relative abundance of *Niastella* bacteria. All currently known species in this genus have been isolated from soil, and may be grown on R2A agar plates (Weon *et al.*, 2006; Zhang *et al.*, 2010; Kim *et al.*, 2015). The genus *Niastella* comprises six species, two of which (*Niastella koreensis* and *Niastella yeongjuensis*) were isolated from soil cultivated with ginseng (Weon *et al.*, 2006). However, Vendan *et al.* (2010) failed to isolate a *Niastella* bacterium from ginseng tissue. These findings and the present results suggest that plant-associated *Niastella* is recalcitrant to isolation with standard R2A medium.

### Clustering analysis of 16S rRNA gene sequences of clones and isolates and selection of sugar beet growth-promoting bacteria

**Fig. 3.**
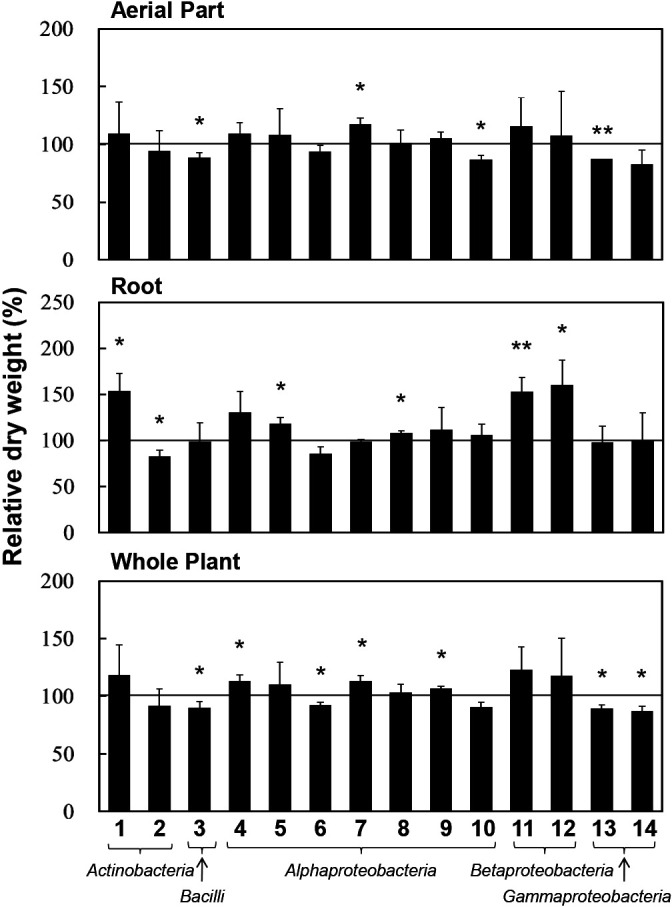
Inoculation effects of sugar beet lateral root-associated bacteria on sugar beet seedling growth. Lateral root-associated bacteria were inoculated onto sugar beet seedlings grown in a pot, and the dry weights of seedlings were measured after 4‍ ‍weeks of cultivation. Control plants were inoculated with sterilized water. Twelve seedlings in a tray were used in an inoculation test with each isolate, and this test was repeated three or four times at different dates to ensure the reproducibility of PGP effects. The dry weights of inoculated seedlings were compared to those of control seedlings (non-inoculated seedlings) by Welch’s *t*-test (two-tailed). Error bars indicate the standard deviation. Single and double asterisks indicate a significant difference at *P*<0.05 and *P*<0.01, respectively. Bacterial isolates: 1, *Nocardioides* sp. RP110; 2, *Streptomyces* sp. TP071; 3, *Bacillus* sp. TP182; 4, *Asticcacaulis* sp. RK043; 5, *Mesorhizobium* sp. TP027; 6, *Neorhizobium* sp. RK064; 7, *Sphingobium* sp. RK166; 8, *Sphingomonas* sp. RP195; 9, *Sphingopyxis* sp. RK106; 10, *Tardiphaga* sp. RK140; 11, *Polaromonas* sp. RK103; 12, *Variovorax* sp. RK170; 13, *Pantoea* sp. RK126; 14, *Rhizobacter* sp. RK021.

A total of 2,645 sequences of the 16S rRNA gene from culture-independent clones (1,980 clones) and culture-dependent isolates (665 isolates) were clustered into 456 OTUs at a sequence identity of 97% (Table S3). Among 279 lateral root-relating OTUs, 93 consisted of only isolates, while 30 contained both clone and isolate sequences. Among the remaining 156 OTUs, no isolates were obtained in the present study (Table S3). Based on the criteria for the selection of OTUs, such as the specificity and stability of colonization to a tissue and phylogenetic novelty, the representative isolates of 44 OTUs were selected (Table S4) as candidate PGPBs and inoculated onto the seedlings of sugar beet. The results obtained revealed eight and six isolates as plant growth-promoting and growth-inhibiting bacteria, respectively (Fig. 3 and S1). BLAST search analyses using 16S rRNA gene sequences indicated that two isolates exerting plant growth-promoting effects (*Asticcacaulis* sp. RK043 and *Mesorhizobium* sp. TP027) are potential novel species based on their identity to known species (Table 4). Furthermore, phylogenetic tree analyses suggested that three isolates (*Asticcacaulis* sp. RK043, *Mesorhizobium* sp. TP027, and *Rhizobacter* sp. RK021) are novel species based on their phylogenetic positions (Fig. S2, S3, and S4).

*Variovorax* are typical phylogenetic groups of PGPBs for a wide range of plant species, including sugar beet (Zhou *et al.*, 2017). Seven genera (*Asticcacaulis*, *Mesorhizobium*, *Nocardioides*, *Sphingobium*, *Sphingomonas*, *Sphingopyxis*, and *Polaromonas*) were newly identified as PGPBs for sugar beet, and two (*Asticcacaulis* and *Polaromonas*) were demonstrated to exert growth-promoting effects on a plant for the first time.

The selection of PGPBs has often been conducted based on known PGPTs, such as nitrogen fixation and IAA production. However, the examination of known PGPTs is a time-consuming and laborious task that has been the bottleneck for the large-scale selection of PGPBs. More importantly, possession of the activity of a known PGPT does not always guarantee that a PGPB will exert growth-promoting effects in inoculation tests (Barazani and Friedman, 1999; Cardinale *et al.*, 2015). In contrast, in the present study, large-scale isolate collections were initially constructed from target tissues via random isolation, and the candidate isolates of PGPBs were then selected based on 16S rRNA gene sequence data. Clustering analyses using the combined sequence data of clone libraries and isolate collections proposed four criteria (the novelty of the sequence, relative abundance and persistence in a target tissue, and its identity to known beneficial or nonpathogenic bacteria) as an indication for selecting candidate isolates of PGPB (Table S4). The ability to colonize a plant tissue is an important trait for PGPBs (Lugtenberg and Kamilova, 2009; Quecine *et al.*, 2012), and the relative abundance of an OTU in a tissue is considered to reflect its compatibility and persistence in a plant tissue.

In addition, deleterious bacteria for sugar beet seedlings were identified as growth-inhibiting bacteria in the present study (*Bacillus* sp. TP182, *Neorhizobium* sp. RK064, *Pantoea* sp. RK126. *Rhizobacter* sp. RK021, *Tardiphaga* sp. RK140, and *Streptomyces* sp. TP071 in Table 4). *Bacillus* spp. are generally considered to be beneficial bacteria because of their growth-promoting effects on many plants, including sugar beet (Çakmakçi *et al.*, 2006; Shi *et al.*, 2010; Park *et al.*, 2017). The present results and previous findings revealed the difficulty of selecting PGPB based solely on phylogenetic information, and indicate the importance of an inoculation test to screen PGPBs on plants. Based on the relative abundance of OTUs in root tissues, deleterious bacteria are considered to exhibit a competitive colonization ability relative to PGPBs (Table 5). The characterization and ecological control of deleterious bacteria are also important for maximizing the effects of PGPBs. Deleterious bacteria may have the ability to interfere with the beneficial effects of PGPBs on plant tissue, and in addition to several physiochemical factors and the genetic background of crops, this interference may partially explain why the effects of PGPBs are often unstable, even under practical field conditions (Timmusk *et al.*, 2005).

## Conclusion

The present study revealed some of the characteristics of the phylogenetic composition of sugar beet-associated bacteria and identified eight isolates of novel PGPBs and six isolates of deleterious bacteria for sugar beet at the species level. The majority of these PGPBs belonging to seven genera (*Nocardioides*, *Asticcacaulis*, *Mesorhizobium*, *Sphingobium*, *Sphingomonas*, *Sphingopyxis*, and *Polaromonas*) were newly identified as PGPBs for sugar beet at the genus level, and two isolates belonging to two genera (*Asticcacaulis* and *Polaromonas*) were identified as PGPBs on a plant at the genus level for the first time. These results demonstrated that a community analysis-based selection is a highly efficient strategy for the initial selection of PGPBs in combination with large-scale isolate collections that increases the likelihood of identifying novel PGPBs. Further analyses of the biochemical and ecological characteristics of beneficial/deleterious bacteria isolated in the present study will provide a more detailed understanding of plant-microbe interactions under field conditions and possibly facilitate the utilization of these beneficial bacteria in agricultural practice for reducing chemical use in sugar beet production.

## Citation

Okazaki, K., Tsurumaru, H., Hashimoto, M., Takahashi, H., Okubo, T., Ohwada, T., et al. (2021) Community Analysis-based Screening of Plant Growth-promoting Bacteria for Sugar Beet. *Microbes Environ ***36**: ME20137.

https://doi.org/10.1264/jsme2.ME20137

## Supplementary Material

Supplementary Material

## Figures and Tables

**Table 1.** Alpha diversity indices of 16S rRNA gene sequences for clone libraries and isolate collections derived from sugar beet-associated bacteria

Libraries/Collections		Clone libraries																Isolate collections				
Tissues		Leaf blade (LB)				Petiole (PE)				Tap root (TR)				Lateral root (LR)				Lateral root (LR)				
Experimental fields		NPK	PK	K		NPK	PK	K		NPK	PK	K		NPK	PK	K		PK			K	
Isolation media		—	—	—		—	—	—		—	—	—		—	—	—		R2A	TSA		R2A	TSA
Library/collection names		LB-NPK	LB-PK	LB-K		PE-NPK	PE-PK	PE-K		TR-NPK	TR-PK	TR-K		LR-NPK	LR-PK	LR-K		LR-PK-R	LR-PK-T		LR-K-R	LR-K-T
Statistics																						
No. of sequences		175	177	167		145	178	178		152	164	178		180	135	151		171	148		180	166
No. of OTUs^a^		42	37	35		40	35	31		70	72	71		97	75	85		52	48		54	48
No. of singletons		20	14	16		17	18	12		47	49	42		61	52	58		28	24		30	28
Library coverage (%)^b^		88.6	92.1	90.4		88.3	89.9	93.3		69.1	70.1	76.4		66.1	61.5	61.6		83.6	83.8		83.3	83.1
Diversity indexes																						
Chao1		63	48	55		59	73	44		224	170	133		199	186	212		115	73		163	86
ACE		105	49	76		59	90	52		164	291	197		318	300	378		124	102		148	97
Shannon index (*H’*)		3.1	2.9	2.9		3.2	2.9	2.6		3.7	3.6	3.7		4.3	4.0	4.2		3.4	3.3		3.5	3.1
Simpson index (1/*D*)		15.3	12.5	11.9		17.9	13.6	8.0		25.8	16.1	25.1		79.0	54.8	67.0		21.3	15.5		26.1	11.8

^a^ OTUs were defined at 97% sequence identity. ^b^ Library coverage Cx was calculated as follows: Cx=1–(*n*/N), where *n* is the number of singletons that are encountered only once in a library or collection, and N is the total number of clones or isolates.

**Table 2.** Relative abundance of major taxa for clone libraries or isolate collections derived from sugar beet-associated bacteria^a^

Libraries/Collections		Clone libraries (%)																Isolate collections (%)				
Tissues		Leaf blade				Petiole				Tap root				Lateral root				Lateral root				
Experimental fields		NPK	PK	K		NPK	PK	K		NPK	PK	K		NPK	PK	K		PK			K	
Isolation medium		—	—	—		—	—	—		—	—	—		—	—	—		R2A	TSA		R2A	TSA
Library/collection name		LB-NPK	LB-PK	LB-K		PE-NPK	PE-PK	PE-K		TR-NPK	TR-PK	TR-K		LR-NPK	LR-PK	LR-K		LR-PK-R	LR-PK-T		LR-K-R	LR-K-T
Phylum																						
*Acidobacteria*		—	—	—		—	—	—		—	—	—		3.9	2.2	4.0		—	—		—	—
*Actinobacteria*		5.1	7.9	5.4		9.7	2.2	2.8		7.2	6.1	3.9		6.7	1.5	4.6		34.5	52.0		14.4	42.2
*Bacteroidetes*		—	—	—		—	—	—		—	—	—		26.1	19.3	19.2		1.2	—		3.9	—
*Firmicutes*		6.9	4.0	1.2		8.3	2.2	1.1		2.6	0.6	1.1		0.6	—	1.3		—	3.4		0.6	3.6
*Planctomycetes*		—	0.6	0.6		—	—	—		4.6	4.3	6.7		2.8	0.7	1.3		—	—		—	—
*Proteobacteria*		88.0	87.6	92.2		81.4	95.5	94.4		79.6	86.0	83.1		56.1	69.6	61.6		64.3	44.6		81.1	54.2
*Verrucomicrobia*		—	—	—		—	—	—		4.6	2.4	2.2		1.7	1.5	4.0		—	—		—	—
Others		—	—	0.6		0.7	—	1.7		1.3	0.6	2.8		2.2	5.2	4.0		—	—		—	—
Class																					
*Actinobacteria*		5.1	7.9	5.4		9.7	2.2	2.8		7.2	6.1	3.9		6.7	1.5	4.6		34.5	52.0		14.4	42.2
*Alphaproteobacteria*		29.1	41.2	22.8		57.2	58.4	69.7		46.7	59.8	59.6		15.6	25.9	23.2		52.0	29.7		61.1	27.7
*Bacilli*		6.9	2.8	1.2		8.3	2.2	1.1		2.6	0.6	1.1		—	—	0.7		—	3.4		0.6	3.6
*Betaproteobacteria*		2.9	1.1	10.2		9.0	2.8	2.2		13.2	6.7	9.6		13.3	9.6	14.6		1.8	2.7		9.4	—
*Deltaproteobacteria*		—	—	—		—	—	—		—	1.2	0.6		3.9	1.5	2.0		—	—		—	—
*Gammaproteobacteria*		56.0	45.2	59.3		15.2	34.3	22.5		19.7	17.7	11.8		22.8	31.9	21.2		10.5	12.2		10.6	26.5
*Planctomycetia*		—	0.6	0.6		—	—	—		4.6	4.3	6.7		2.8	0.7	1.3		—	—		—	—
*Spartobacteria*		—	—	—		—	—	—		3.9	1.8	1.1		—	—	0.7		—	—		—	—
*Sphingobacteriia*		—	—	—		—	—	—		—	—	—		23.3	17.8	16.6		1.2	—		1.1	—
Others		—	1.1	0.6		0.7	—	1.7		2.0	1.8	5.6		11.7	11.1	15.2		—	—		2.8	—
Order																						
*Actinomycetales*		5.1	7.9	5.4		9.7	1.1	2.8		7.2	6.1	3.9		6.7	0.7	4.6		34.5	52.0		14.4	42.2
*Bacillales*		4.6	0.6	—		7.6	0.6	1.1		2.6	0.6	1.1		—	—	0.7		—	3.4		0.6	3.6
*Burkholderiales*		2.9	1.1	10.2		9.0	2.8	2.2		13.2	6.7	9.6		12.2	8.1	12.6		1.8	2.7		9.4	—
*Caulobacterales*		—	—	—		—	—	—		0.7	0.6	0.6		2.2	2.2	1.3		4.7	1.4		1.1	—
*Enterobacteriales*		33.1	28.8	41.3		9.0	19.1	12.9		0.7	—	1.1		1.1	—	1.3		0.6	1.4		2.2	19.9
*Legionellales*		—	—	—		—	—	0.6		3.9	3.0	0.6		—	—	—		—	—		—	—
*Planctomycetales*		—	0.6	0.6		—	—	—		4.6	4.3	6.7		2.8	0.7	1.3		—	—		—	—
*Pseudomonadales*		22.3	15.8	15.0		4.8	10.1	6.7		2.6	2.4	2.8		8.9	11.1	7.9		2.9	4.7		4.4	3.6
*Rhizobiales*		25.1	36.7	21.0		44.8	48.9	63.5		28.3	35.4	38.8		8.3	11.9	11.3		28.1	25.7		33.3	26.5
*Sphingobacteriales*		—	—	—		—	—	—		—	—	—		23.3	17.8	16.6		1.2	0.0		1.1	—
*Sphingomonadales*		4.0	2.8	1.8		11.7	9.6	4.5		16.4	23.2	18.5		3.9	11.1	9.9		19.3	2.7		25.0	1.2
*Xanthomonadales*		0.6	0.6	2.4		1.4	4.5	1.7		2.0	0.6	1.1		4.4	14.8	3.3		7.0	6.1		3.9	3.0
Unclassified *Gammaproteobacteria*		—	—	0.6		—	0.6	0.6		10.5	11.6	6.2		5.6	5.9	7.9		—	—		—	—
Others		2.3	5.1	1.8		2.1	2.8	3.4		7.2	5.5	9.0		20.6	15.6	21.2		—	—		4.4	—
Family																						
*Bacillaceae 1*		3.4	—	—		2.8	—	1.1		1.3	—	0.6		—	—	0.7		—	2.7		0.6	3.0
*Bradyrhizobiaceae*		—	—	0.6		—	0.6	—		7.2	7.9	15.7		1.7	3.0	1.3		1.8	1.4		2.2	3.0
*Burkholderiaceae*		—	—	4.8		2.1	—	—		4.6	2.4	1.7		2.2	0.7	0.0		1.2	—		—	—
*Caulobacteraceae*		—	—	—		—	—	—		0.7	0.6	0.6		2.2	2.2	1.3		4.7	1.4		1.1	—
*Chitinophagaceae*		—	—	—		—	—	—		—	—	—		21.1	17.0	15.2		—	—		0.6	—
*Comamonadaceae*		1.1	1.1	4.8		6.2	2.8	2.2		3.3	1.8	1.7		4.4	3.0	8.6		—	0.7		8.9	—
*Enterobacteriaceae*		33.1	28.8	41.3		9.0	19.1	12.9		0.7	—	1.1		1.1	—	1.3		0.6	1.4		2.2	19.9
*Hyphomicrobiaceae*		—	—	—		0.7	—	—		1.3	1.2	4.5		1.7	0.7	2.0		4.1	8.1		5.0	7.8
*Methylobacteriaceae*		15.4	29.9	14.4		24.1	32.6	45.5		—	—	0.6		—	—	—		—	—		—	—
*Microbacteriaceae*		1.1	2.8	0.6		0.7	—	—		0.7	0.6	0.6		—	—	—		8.8	18.9		1.1	3.0
*Micrococcaceae*		3.4	2.8	4.2		4.8	0.6	2.2		—	—	—		—	—	—		1.2	0.7		1.1	3.0
*Mycobacteriaceae*		0.6	—	0.6		2.8	0.6	0.0		2.6	1.8	1.7		—	—	—		1.2	—		—	1.2
*Nocardioidaceae*		—	1.1	—		—	—	0.6		—	—	—		—	—	—		4.1	0.7		1.1	—
*Oxalobacteraceae*		1.7	—	0.6		0.7	—	—		3.3	1.8	5.6		5.6	4.4	4.0		—	—		0.6	—
*Phyllobacteriaceae*		5.1	2.8	0.6		13.8	10.1	9.6		3.3	6.7	7.3		0.6	0.0	2.0		10.5	6.1		6.7	1.2
*Planctomycetaceae*		—	0.6	0.6		—	—	—		4.6	4.3	6.7		2.8	0.7	1.3		—	—		—	—
*Pseudomonadaceae*		21.7	15.8	15.0		4.8	9.6	6.7		2.6	2.4	2.8		8.9	11.1	7.9		2.9	4.7		4.4	3.6
*Rhizobiaceae*		2.3	3.4	5.4		5.5	3.4	6.7		15.8	17.1	9.6		3.9	6.7	4.6		11.1	9.5		18.3	13.9
*Sphingomonadaceae*		4.0	2.8	1.8		11.7	9.6	4.5		16.4	23.2	18.5		3.9	11.1	9.9		19.3	2.7		24.4	1.2
*Staphylococcaceae*		—	—	—		4.1	0.6	—		—	—	—		—	—	—		—	—		—	—
*Streptomycetaceae*		—	—	—		—	—	—		2.6	2.4	1.1		5.0	0.7	4.6		16.4	25.7		9.4	33.1
*Xanthomonadaceae*		0.6	0.6	2.4		1.4	4.5	1.7		2.0	0.6	1.1		4.4	14.1	3.3		7.0	6.1		3.9	3.0
Others		6.3	7.3	2.4		4.8	6.2	6.2		27.0	25.0	18.5		30.6	24.4	31.8		5.3	9.5		8.3	3.0

^a^ Gray indicates the taxa with a significant difference (*P*<0.05) to the NPK field library in each tissue.

**Table 3.** Relative abundance of major genera for clone libraries or isolate collections derived from sugar beet-associated bacteria^a^

Libraries/Collections		Clone libraries (%)																Isolate collections (%)				
Tissues		Leaf blade				Petiole				Tap root				Lateral root				Lateral root				
Experimental fields		NPK	PK	K		NPK	PK	K		NPK	PK	K		NPK	PK	K		PK			K	
Isolation media		—	—	—		—	—	—		—	—	—		—	—	—		R2A	TSA		R2A	TSA
Library/collection names		LB-NPK	LB-PK	LB-K		PE-NPK	PE-PK	PE-K		TR-NPK	TR-PK	TR-K		LR-NPK	LR-PK	LR-K		LR-PK-R	LR-PK-T		LR-K-R	LR-K-T
Genus																						
*Arthrobacter*		2.9	2.8	4.2		4.8	0.6	2.2		—	—	—		—	—	—		0.6	—		0.6	3.0
*Bacillus*		2.9	—	—		2.8	—	1.1		1.3	—	0.6		—	—	0.7		—	2.7		0.6	3.0
*Curtobacterium*		—	2.8	—		—	—	—		—	—	—		—	—	—		—	—		—	—
*Devosia*		—	—	—		—	—	—		1.3	1.2	4.5		1.1	0.7	2.0		4.1	8.1		5.0	7.8
*Enterobacter*		—	—	3.0		—	—	—		—	—	—		—	—	—		—	—		—	—
*Mesorhizobium*		—	—	—		—	—	—		2.6	5.5	6.7		—	—	2.0		6.4	—		6.1	—
*Methylobacterium*		15.4	29.9	14.4		24.1	32.6	45.5		—	—	0.6		—	—	—		—	—		—	—
*Microbacterium*		0.6	—	—		—	—	—		—	0.6	—		—	—	—		5.3	16.9		1.1	1.8
*Mycobacterium*		0.6	—	0.6		2.8	0.6	—		2.6	1.8	1.7		—	—	—		1.2	—		—	1.2
*Neorhizobium*		—	—	—		—	—	—		—	1.2	1.7		2.2	3.7	2.0		2.9	—		7.8	—
*Niastella*		—	—	—		—	—	—		—	—	—		11.7	11.1	7.9		—	—		—	—
*Nocardioides*		—	—	—		—	—	0.6		—	—	—		—	—	—		4.1	—		1.1	—
*Novosphingobium*		—	—	—		—	0.6	—		15.8	22.0	14.0		3.3	8.1	4.6		1.8	2.0		10.6	1.2
*Pantoea*		5.1	2.3	6.6		2.8	—	5.6		—	—	1.1		1.1	—	0.7		—	1.4		—	4.8
*Phyllobacterium*		5.1	2.8	0.6		13.8	10.1	9.6		—	1.2	0.6		—	—	—		0.6	2.7		—	0.6
*Polaromonas*		—	—	—		—	—	—		2.6	1.2	0.6		2.2	1.5	3.3		—	—		7.2	—
*Pseudomonas*		21.7	15.8	15.0		4.8	9.6	6.7		2.6	2.4	2.8		7.2	7.4	6.0		2.9	4.7		3.9	3.0
*Ralstonia*		—	—	3.6		1.4	—	—		—	—	—		—	—	—		—	—		—	—
*Rhizobium*		2.3	3.4	4.8		5.5	3.4	6.7		15.1	15.2	7.9		1.7	3.0	2.6		7.0	9.5		9.4	13.3
*Sphingomonas*		4.0	2.8	1.8		11.7	9.0	4.5		—	—	—		0.6	1.5	1.3		13.5	—		7.2	—
*Streptomyces*		—	—	—		—	—	—		2.6	2.4	1.1		5.0	0.7	4.6		16.4	24.3		9.4	33.1
*Variovorax*		1.1	1.1	4.8		6.2	2.8	1.7		0.7	0.6	0.6		1.7	—	—		—	0.7		1.1	—
*Yersinia*		2.9	—	—		—	—	—		—	—	—		—	—	—		—	—		—	—
Unclassified *Bradyrhizobiaceae*		—	—	—		—	—	—		0.7	1.2	5.1		—	1.5	0.7		—	—		0.6	—
Unclassified *Chitinophagaceae*		—	—	—		—	—	—		—	—	—		7.2	2.2	6.0		—	—		—	—
Unclassified *Enterobacteriaceae*		22.3	20.9	28.1		4.1	16.9	5.1		0.7	—	—		—	—	0.7		—	—		1.1	10.8
Others		13.1	15.3	12.6		15.2	14.0	10.7		51.3	43.3	50.6		55.0	58.5	55.0		33.3	27.0		27.2	16.3

^a^ Gray highlight indicates taxa with a significant difference (*P*<0.05) from the NPK field library in each tissue.

**Table 4.** BlastN search results with nearly the full sequence of the 16S rRNA gene of growth-promoting and -inhibiting bacteria for sugar beet seedlings

OTU No.	Isolate name	Inoculation effect^a^	BlastN search results^b^	
			Closest known species	Identity (%)
	*Actinobacteria*			
OTU-301	*Nocardioides* sp. RP110 (LC040866)^c^	+	*Nocardioides cavernae* (NR_156135)	100
OTU-272	*Streptomyces* sp. TP071 (LC602158)	–	*Streptomyces mirabilis* (EF371431)	100
	*Bacilli*			
OTU-329	*Bacillus* sp. TP182 (LC602159)	–	*Bacillus gibsonii* (FJ937920)	100
	*Alphaproteobacteria*			
OTU-226	*Asticcacaulis* sp. RK043 (LC040869)	+	*Asticcacaulis benevestitus* (NR_042433)	98
OTU-170	*Mesorhizobium* sp. TP027 (LC040873)	+	*Mesorhizobium chacoense* (NR_025411)	98
OTU-166	*Neorhizobium* sp. RK064 (LC602160)	–	*Neorhizobium galegae* (HG938355)	100
OTU-189	*Sphingobium* sp. RK166 (LC602162)	+	*Sphingobium aromaticiconvertens* (MF101093)	100
OTU-191	*Sphingomonas* sp. RP195 (LC602164)	+	*Sphingomonas asaccharolytica* (NR_029327)	100
OTU-199	*Sphingopyxis* sp. RK106 (LC602163)	+	*Sphingopyxis taejonensis* (NR_024999)	100
OTU-218	*Tardiphaga* sp. RK140 (LC602161)	–	*Tardiphaga robiniae* (CP050292)	99
	*Betaproteobacteria*			
OTU-87	*Polaromonas* sp. RK103 (LC040879)	+	*Polaromonas ginsengisoli* (AB245355)	100
OTU-86	*Variovorax* sp. RK170 (LC040880)	+	*Variovorax paradoxus* (CP002417)	100
	*Gammaproteobacteria*			
OTU-69	*Pantoea* sp. RK126 (LC602165)	–	*Pantoea ananatis *(CP020943)	100
OTU-96	*Rhizobacter* sp. RK021 (LC040878)	–	*Methylibium petroleiphilum* (CP000555)	99

^a^ “+” and “–” indicate growth-promoting and -inhibiting bacteria, respectively. ^b^ Results with approximately 1,400 bp using the NCBI database (https://blast.ncbi.nlm.nih.gov/Blast.cgi) are shown. ^c^ The numbers in parentheses indicate the accession number.

**Table 5.** Relative abundance of OTUs in clone libraries of 16S rRNA genes derived from sugar beet-associated bacteria

Tissues		Leaf blade				Petiole				Tap root				Lateral root			Isolation
Experimental fields		NPK	PK	K		NPK	PK	K		NPK	PK	K		NPK	PK	K	
**Growth-inhibiting bacteria**																	
OTU-69		—	—	—		—	—	—		—	—	—		—	—	—	*Pantoea* sp. RK126 (LC602165)
OTU-96		—	—	—		—	—	—		—	—	—		0.6	4.4	2.0	*Rhizobacter* sp. RK021 (LC040878)
OTU-166		—	—	—		—	—	—		—	1.2	1.7		2.2	3.7	2.0	*Neorhizobium* sp. RK064 (LC602160)
OTU-218		—	—	—		—	—	—		2.6	1.8	4.5		0.6	0.7	—	*Tardiphaga* sp. RK140 (LC602161)
OTU-272		—	—	—		—	—	—		—	—	—		1.7	0.7	0.7	*Streptomyces* sp. TP071 (LC602158)
OTU-329		1.7	—	—		2.8	—	—		1.3	—	0.6		—	—	—	*Bacillus* sp. TP182 (LC602159)
**Growth-promoting bacteria**																	
OTU-86		1.1	1.1	4.8		6.2	2.8	1.7		0.7	0.6	0.6		1.7	—	—	*Variovorax* sp. RK170 (LC040880)
OTU-87		—	—	—		—	—	—		2.6	1.2	0.6		2.2	1.5	3.3	*Polaromonas* sp. RK103 (LC040879)
OTU-170		—	—	—		—	—	—		—	—	—		—	—	—	*Mesorhizobium* sp. TP027 (LC040873)
OTU-189		—	—	—		—	—	—		0.7	—	2.8		—	1.5	2.0	*Sphingobium* sp. RK166 (LC602162)
OTU-191		—	—	—		—	—	—		—	—	—		0.6	1.5	1.3	*Sphingomonas* sp. RP195 (LC602164)
OTU-199		—	—	—		—	—	—		—	—	—		—	—	—	*Sphingopyxis* sp. RK106 (LC602163)
OTU-226		—	—	—		—	—	—		—	—	—		—	0.7	—	*Asticcacaulis* sp. RK043 (LC040869)
OTU-301		—	—	—		—	—	—		—	—	—		—	—	—	*Nocardioides* sp. RP110 (LC040866)
