# Supplementary material for: Community Analysis-based Screening of Plant Growth-promoting Bacteria for Sugar Beet
Source: Microbes Environ. 2021 Apr 27;36(2):ME20137. doi: 10.1264/jsme2.ME20137 (PMC8209457; doi:10.1264/jsme2.ME20137)
Supplement: Supplementary file 1 — Supplementary Material [file 36_20137_s1.pdf]

Supplementary materials

Title: Community analysis-based screening of plant growth-promoting bacteria for sugar beet

Authors: Kazuyuki Okazaki[1], Hirohito Tsurumaru[2], Megumi Hashimoto[2], Hiroyuki Takahashi[1], Takashi Okubo[2], Takuji Ohwada[3], Kiwamu Minamisawa[2] and Seishi Ikeda[1]*

Author affiliation: [1]Memuro Research Station, Hokkaido Agricultural Research Center, National Agriculture and Food Research Organization, 9-4 Shinsei-minami, Memuro, Kasai-gun, Hokkaido 082-0081, Japan; [2]Graduate School of Life Science, Tohoku University, 2-1-1 Katahira, Aoba-ku, Sendai, Miyagi 980-8577, Japan; [3]Department of Agricultural and Life Sciences, Obihiro University of Agriculture and Veterinary Medicine, Obihiro, Hokkaido 080-8555, Japan.

***Corresponding author:** Seishi Ikeda, Memuro Research Station, Hokkaido Agricultural Research Center, National Agriculture and Food Research Organization, 9-4 Shinsei-minami, Memuro, Kasai-gun, Hokkaido 082-0081, Japan. E-mail: sikeda67@affrc.go.jp; Tel.: +81-155-62-9276; Fax: +81-155-61-2127.

Table S1. List of accession numbers of nucleotide sequences

Library / collection names	No. of sequences	Accession No. Beginig	-	End
LB-NPK	176	LC038237	-	LC038411
LB-PK	177	LC038412	-	LC038588
LB-K	167	LC038589	-	LC038755
PE-NPK	145	LC038756	-	LC038900
PE-PK	178	LC038901	-	LC039078
PE-K	178	LC039079	-	LC039256
LR-NPK	180	LC039257	-	LC039436
LR-PK	135	LC039437	-	LC039571
LR-K	151	LC039572	-	LC039722
TR-NPK	152	LC039723	-	LC039874
TR-PK	164	LC039875	-	LC040038
TR-K	178	LC040039	-	LC040216
LR-PK-R	169	LC040217	-	LC040385
LR-K-R	171	LC040386	-	LC040556
LR-PK-T	145	LC040557	-	LC040701
LR-K-T	163	LC040702	-	LC040864

Table S2. Chemical characteristics of soils in experimental fields of the present study

Experimental fields	pH (H2O)	P_2O_5 ($mg·kg^{-1}$)	K_2O ($mg·kg^{-1}$)	M_gO ($mg·kg^{-1}$)	C_aO ($mg·kg^{-1}$)	Total nitrogen (%)	NO_3-N ($mg·kg^{-1}$)	NH_4-N ($mg·kg^{-1}$)	Phosphate absorption coefficient	CEC ($mg·kg^{-1}$)
NPK	5.4	106	181	135	1,498	0.27	36	5.5	1,812	196
PK	5.9	144	237	229	2,319	0.27	52	5.7	1,678	208
K	5.8	50	215	191	1,560	0.26	35	5.5	1,737	180

Table S3. Relative abundance of OTUs in clone libraries or isolate collections of the 16S rRNA genes derived from sugar beet-associated bacteria [a]

Libraries / Collections	Clone libraries (%)												Isolate collections (%)				Closest relatives in known species	Acc. No.	Identity (%)
Tissues	Leaf blade			Petiole			Tap root			Lateral root			Lateral root						
Experimental fields	NPK	PK	K	NPK	PK	K	NPK	PK	K	NPK	PK	K	PK		K				
Isolation medium	-	-	-	-	-	-	-	-	-	-	-	-	R2A	TSA	R2A	TSA			
Library / Collection name	LB-NPK	LB-PK	LB-K	PE-NPK	PE-PK	PE-K	TR-NPK	TR-PK	TR-K	LR-NPK	LR-PK	LR-K	LR-PK-R	LR-PK-T	LR-K-R	LR-K-T			
OTU-1	-	-	-	-	-	-	2.0	-	-	-	-	-	-	-	-	-	*Legionella drancourtii*	NR_026335	88
OTU-2	-	-	-	-	-	-	-	1.2	-	-	-	-	-	-	-	-	*Legionella hackeliae*	NR_104894	88
OTU-3	-	-	-	-	-	-	2.0	-	2.2	-	-	-	-	-	-	-	*Legionella hackeliae*	NR_104894	87
OTU-4	-	-	-	-	-	-	1.3	1.8	1.1	-	-	-	-	-	-	-	*Legionella clemsonensis*	CP016397	88
OTU-5	-	-	-	-	-	-	-	0.6	-	-	-	-	-	-	-	-	*Legionella clemsonensis*	KX694517	88
OTU-6	-	-	-	-	-	-	0.7	-	-	-	-	-	-	-	-	-	*Legionella hackeliae*	NR_104894	88
OTU-7	-	-	-	-	-	-	-	0.6	-	-	-	-	-	-	-	-	*Legionella clemsonensis*	CP016397	88
OTU-8	-	-	-	-	-	-	-	0.6	-	-	-	-	-	-	-	-	*Legionella hackeliae*	NR_104894	88
OTU-9	-	-	-	-	-	-	-	0.6	-	-	-	-	-	-	-	-	*Legionella hackeliae*	NR_104894	87
OTU-10	-	-	-	-	-	-	-	0.6	-	-	-	-	-	-	-	-	*Legionella hackeliae*	NR_104894	88
OTU-11	-	-	-	-	-	-	0.7	-	-	-	-	-	-	-	-	-	*Legionella cardiaca*	NR_109426	88
OTU-12	-	-	-	-	-	-	0.7	-	-	-	-	-	-	-	-	-	*Aquicella siphonis*	NR_025764	91
OTU-13	-	-	-	-	-	-	-	0.6	-	-	-	-	-	-	-	-	*Aquicella siphonis*	AY359284	92
OTU-14	-	-	-	-	-	-	0.7	-	-	-	-	-	-	-	-	-	*Aquicella siphonis*	NR_025764	92
OTU-15	-	-	-	-	-	-	-	0.6	-	-	-	-	-	-	-	-	*Aquicella siphonis*	NR_025764	94
OTU-16	-	-	-	-	-	-	-	0.6	-	-	-	-	-	-	-	-	*Aquicella siphonis*	NR_025764	92
OTU-17	-	-	-	-	-	-	0.7	-	-	-	-	-	-	-	-	-	*Aquicella siphonis*	NR_025764	92
OTU-18	-	-	-	-	-	-	-	0.6	0.6	-	-	-	-	-	-	-	*Rickettsiella grylli*	RGU97547	97
OTU-19	-	-	-	-	-	-	0.7	-	-	-	-	-	-	-	-	-	*Legionella norrlandica*	KU508790	97
OTU-20	-	-	-	-	-	-	-	1.2	0.6	-	-	-	-	-	-	-	*Legionella worsleiensis*	NR_044971	97
OTU-21	-	-	-	-	-	-	0.7	-	-	-	-	-	-	-	-	-	*Legionella worsleiensis*	NR_044971	95
OTU-22	-	-	-	-	-	-	0.7	-	-	-	-	-	-	-	-	-	*Legionella drozanskii*	NR_036803	96
OTU-23	-	-	-	-	-	0.6	-	-	-	-	-	-	-	-	-	-	*Legionella donaldsonii*	KM504126	96
OTU-24	-	-	-	-	-	-	2.0	-	-	-	-	-	-	-	-	-	*Legionella pneumophila*	FR799702	90
OTU-25	-	-	0.6	-	-	-	-	-	-	-	-	-	-	-	-	-	*Ca.* Berkiella cookevillensis	EF492067	88
OTU-26	-	-	-	-	-	-	-	0.6	-	-	-	-	-	-	-	-	*Methylococcus capsulatus*	KF952440	87
OTU-27	-	-	-	-	-	-	-	0.6	-	-	-	-	-	-	-	-	*Legionella clemsonensis*	CP016397	88
OTU-28	-	-	-	-	-	-	-	0.6	-	-	-	-	-	-	-	-	*Legionella clemsonensis*	CP016397	87
OTU-29	-	-	-	-	-	-	-	-	-	-	0.7	-	-	-	-	-	*Ca.* Ovatusbacter abovo	LN875063	92
OTU-30	-	-	-	-	-	-	0.7	-	-	-	-	-	-	-	-	-	*Ca.* Ovatusbacter abovo	LN875063	94
OTU-31	-	-	-	-	-	-	-	0.6	-	-	-	-	-	-	-	-	*Ca.* Ovatusbacter abovo	LN875063	91
OTU-32	-	-	-	-	-	-	-	1.8	-	-	-	-	-	-	-	-	*Ca.* Ovatusbacter abovo	LN875063	88
OTU-33	-	-	-	-	-	-	0.7	0.6	-	-	-	-	-	-	-	-	*Ca.* Ovatusbacter abovo	LN875063	91
OTU-34	-	-	-	-	-	-	-	-	0.6	-	-	-	-	-	-	-	*Ca.* Berkiella cookevillensis	EF492067	89
OTU-35	-	1.1	-	1.4	-	-	-	-	-	0.6	-	-	-	-	-	-	*Pseudomonas gingeri*	KX817286	99
OTU-36	2.3	2.3	-	-	1.1	-	-	-	-	-	-	-	-	-	-	-	*Pseudomonas endophytica*	NR_136473	99
OTU-37	14.3	11.3	5.4	2.1	1.1	3.9	2.0	1.8	1.7	2.2	5.2	3.3	2.3	4.7	-	1.8	*Pseudomonas extremaustralis*	MF523621	100
OTU-38	-	-	-	-	-	-	-	-	-	-	-	-	-	-	-	0.6	*Pseudomonas mohnii*	MG571738	96
OTU-39	5.1	1.1	9.6	1.4	7.3	2.8	0.7	0.6	1.1	4.4	2.2	2.6	0.6	-	3.9	1.2	*Pseudomonas granadensis*	MG269629	100
OTU-40	-	-	-	-	-	-	-	-	-	0.6	-	-	-	-	-	-	*Burkholderia dabaoshanensis*	NR_133711	90
OTU-41	-	-	-	-	-	-	-	-	-	1.1	0.7	0.7	-	-	-	-	*Cellvibrio japonicus*	NR_074804	92
OTU-42	-	-	-	-	-	-	-	-	-	1.7	0.7	0.7	-	-	-	-	*Pseudohongiella acticola*	NR_136444	91
OTU-43	-	-	-	-	-	-	-	-	-	3.9	3.7	6.0	-	-	-	-	*Pseudomonas psychrotolerans*	KY882086	91
OTU-44	0.6	-	-	-	-	-	-	-	-	-	-	-	-	-	-	-	*Acinetobacter guillouiae*	MF754134	99
OTU-45	-	-	-	-	0.6	-	-	-	-	-	-	-	-	-	-	-	*Moraxella osloensis*	NR_104936	99

Table S3. Continued

Libraries / Collections	Clone libraries (%)												Isolate collections (%)				Closest relatives in known species	Acc. No.	Identity (%)
Tissues	Leaf blade			Petiole			Tap root			Lateral root			Lateral root						
Experimental fields	NPK	PK	K	NPK	PK	K	NPK	PK	K	NPK	PK	K	PK		K				
Isolation medium	-	-	-	-	-	-	-	-	-	-	-	-	R2A	TSA	R2A	TSA			
Library / Collection name	LB-NPK	LB-PK	LB-K	PE-NPK	PE-PK	PE-K	TR-NPK	TR-PK	TR-K	LR-NPK	LR-PK	LR-K	LR-PK-R	LR-PK-T	LR-K-R	LR-K-T			
OTU-46	-	-	-	-	-	-	-	-	-	1.1	-	1.3	-	-	-	-	*Acidibacter ferrireducens*	NR_126260	94
OTU-47	-	-	-	-	0.6	-	-	-	-	-	-	-	-	-	-	-	*Sulfuriflexus mobilis*	NR_152000	91
OTU-48	-	-	-	-	-	-	-	-	-	-	0.7	-	-	-	-	-	*Steroidobacter flavus*	KU195414	93
OTU-49	-	-	-	-	-	-	-	-	-	0.6	-	-	-	-	-	-	*Coxiella burnetii*	CP000890	91
OTU-50	-	-	-	-	-	-	0.7	-	-	-	-	-	-	-	-	-	*Coxiella cheraxi*	NR_116014	92
OTU-51	-	-	-	-	-	-	-	-	1.7	-	-	-	-	-	-	-	*Coxiella burnetii*	CP014561	87
OTU-52	12.0	16.4	22.8	1.4	14.0	2.2	-	-	-	-	-	-	-	-	-	-	*Dickeya chrysanthemi*	EF178670	99
OTU-53	-	-	-	-	0.6	-	-	-	-	-	-	-	-	-	-	-	*Dickeya chrysanthemi*	EF178670	97
OTU-54	-	-	-	0.7	-	-	-	-	-	-	-	-	-	-	-	-	*Dickeya chrysanthemi*	HM016083	97
OTU-55	-	0.6	-	-	-	-	-	-	-	-	-	-	-	-	-	-	*Erwinia chrysanthemi*	HM016083	98
OTU-56	0.6	-	1.2	-	-	-	-	-	-	-	-	-	-	-	-	-	*Cedecea davisae*	KX062011	100
OTU-57	1.7	1.7	1.2	-	-	-	-	-	-	-	-	-	-	-	-	-	*Erwinia toletana*	JX134630	100
OTU-58	-	-	-	0.7	-	-	-	-	-	-	-	-	-	-	-	-	*Escherichia coli*	CP024232	99
OTU-59	0.6	-	3.0	0.7	-	-	-	-	-	-	-	-	0.6	-	-	-	*Enterobacter cloacae*	LC361167	100
OTU-60	-	-	0.6	-	-	-	-	-	-	-	-	-	-	-	-	-	*Citrobacter freundii*	KU570381	97
OTU-61	-	-	1.8	2.1	2.2	1.7	-	-	-	-	-	-	-	-	-	-	*Serratia marcescens*	CP026050	100
OTU-62	-	0.6	-	-	-	-	-	-	-	-	-	-	-	-	-	-	*Erwinia persicina*	KF429292	93
OTU-63	6.9	3.4	-	0.7	2.2	3.4	0.7	-	-	-	-	-	-	-	-	-	*Pantoea dispersa*	KF135238	99
OTU-64	-	-	-	-	-	-	-	-	-	-	-	0.7	-	-	-	-	*Rahnella aquatilis*	KY810674	99
OTU-65	5.1	-	3.6	-	-	-	-	-	-	-	-	-	-	-	-	-	*Yersinia kristensenii*	KJ956650	100
OTU-66	1.1	3.4	-	-	-	-	-	-	-	-	-	-	-	-	-	-	*Hafnia alvei*	MG190854	100
OTU-67	-	-	-	-	-	-	-	-	-	-	-	-	-	-	-	1.2	*Pantoea ananatis*	KM603631	95
OTU-68	-	-	-	-	-	-	-	-	-	-	-	-	-	-	-	0.6	*Pantoea ananatis*	KM603631	96
OTU-69	-	-	-	-	-	-	-	-	-	-	-	-	-	-	2.2	0.6	*Pantoea ananatis*	KX011138	96
OTU-70	-	-	-	-	-	-	-	-	-	-	-	-	-	-	-	1.2	*Pantoea ananatis*	KX118705	96
OTU-71	-	-	-	-	-	-	-	-	-	-	-	-	-	-	-	1.2	*Pantoea ananatis*	KM603631	97
OTU-72	-	-	-	-	-	-	-	-	-	-	-	-	-	-	-	1.8	*Pantoea ananatis*	KM603631	96
OTU-73	-	-	-	-	-	-	-	-	-	-	-	-	-	-	-	0.6	*Pantoea ananatis*	KM603631	96
OTU-74	-	-	-	-	-	-	-	-	-	-	-	-	-	-	-	9.6	*Pantoea ananatis*	KM603631	98
OTU-75	-	-	-	-	-	-	-	-	-	-	-	-	-	1.4	-	-	*Pantoea vagans*	KY816320	97
OTU-76	5.1	2.3	6.6	2.8	-	5.6	-	-	1.1	1.1	-	0.7	-	-	-	-	*Pantoea vagans*	KY127421	100
OTU-77	-	-	-	-	-	-	-	-	-	-	-	-	-	-	-	0.6	*Pantoea ananatis*	KM603631	97
OTU-78	-	-	-	-	-	-	-	-	-	-	-	-	-	-	-	0.6	*Pantoea agglomerans*	MG544110	92
OTU-79	-	-	-	-	-	-	-	-	-	-	-	-	-	-	-	0.6	*Pantoea ananatis*	KX011138	97
OTU-80	-	-	-	-	-	-	-	-	-	-	-	-	-	-	-	0.6	*Flavobacterium acidificum*	MG571690	98
OTU-81	-	-	-	-	-	-	-	-	-	-	-	-	-	-	-	0.6	*Flavobacterium acidificum*	MG571690	97
OTU-82	-	-	0.6	-	-	-	-	-	-	-	-	-	-	-	-	-	*Providencia burhodogranariea*	EU587089	99
OTU-83	-	0.6	-	-	-	-	-	-	-	-	-	-	-	-	-	-	*Morganella morganii*	DQ358125	99
OTU-84	-	-	-	-	-	0.6	-	-	-	-	-	-	-	-	-	-	*Pantoea deleyi*	GQ169377	84
OTU-85	-	-	-	-	-	-	-	-	-	-	-	-	-	-	0.6	-	*Variovorax paradoxus*	KX981413	97
OTU-86	1.1	1.1	4.8	6.2	2.8	1.7	0.7	0.6	0.6	1.7	-	-	-	0.7	0.6	-	*Variovorax paradoxus*	KY486805	100
OTU-87	-	-	-	-	-	-	2.6	1.2	0.6	2.2	1.5	3.3	-	-	7.2	-	*Polaromonas ginsengisoli*	AB245355	100
OTU-88	-	-	-	-	-	-	-	-	-	-	-	0.7	-	-	-	-	*Rhodoferax ferrireducens*	KC855480	97
OTU-89	-	-	-	-	-	-	-	-	-	-	0.7	-	-	-	-	-	*Rhodoferax antarcticus*	AY609198	98
OTU-90	-	-	-	-	-	-	-	-	-	0.6	0.7	1.3	-	-	0.6	-	*Acidovorax radicis*	MF101116	100

Table S3. Continued

Libraries / Collections	Clone libraries (%)												Isolate collections (%)				Closest relatives in known species	Acc. No.	Identity (%)
Tissues	Leaf blade			Petiole			Tap root			Lateral root			Lateral root						
Experimental fields	NPK	PK	K	NPK	PK	K	NPK	PK	K	NPK	PK	K	PK		K				
Isolation medium	-	-	-	-	-	-	-	-	-	-	-	-	R2A	TSA	R2A	TSA			
Library / Collection name	LB-NPK	LB-PK	LB-K	PE-NPK	PE-PK	PE-K	TR-NPK	TR-PK	TR-K	LR-NPK	LR-PK	LR-K	LR-PK-R	LR-PK-T	LR-K-R	LR-K-T			
OTU-91	-	-	-	-	-	0.6	-	-	-	-	-	-	-	-	-	-	*Delftia tsuruhatensis*	KY810708	100
OTU-92	-	-	-	-	-	-	-	-	-	-	-	2.0	-	-	-	-	*Pelomonas saccharophila*	KC914555	99
OTU-93	-	-	-	-	-	-	-	-	-	-	-	0.7	-	-	-	-	*Pelomonas saccharophila*	AB495144	100
OTU-94	-	-	-	-	-	-	-	-	0.6	-	-	0.7	-	-	-	-	*Roseateles depolymerans*	AB495143	100
OTU-95	-	-	-	-	-	-	-	-	-	1.1	-	-	-	-	-	-	*Leptothrix mobilis*	FM886901	96
OTU-96	-	-	-	-	-	-	-	-	-	0.6	4.4	2.0	-	-	0.6	-	*Rhizobacter dauci*	KU341400	98
OTU-97	-	-	-	-	-	-	-	-	0.6	1.7	-	-	-	-	-	-	*Burkholderia terrae*	FJ796430	99
OTU-98	-	-	-	-	-	-	0.7	0.6	0.6	-	-	-	-	-	-	-	*Paraburkholderia phytofirmans*	KF981504	99
OTU-99	-	-	-	-	-	-	-	-	-	-	0.7	-	-	-	-	-	*Burkholderia soli*	KF981587	100
OTU-100	-	-	1.2	-	-	-	-	1.2	-	-	-	-	1.2	-	-	-	*Paraburkholderia fungorum*	MG576015	100
OTU-101	-	-	-	-	-	-	3.3	0.6	0.6	-	-	-	-	-	-	-	*Burkholderia metallica*	MG571741	100
OTU-102	-	-	-	-	-	-	0.7	-	-	-	-	-	-	-	-	-	*Pandoraea pnomenusa*	LN997983	100
OTU-103	-	-	-	0.7	-	-	-	-	-	-	-	-	-	-	-	-	*Ralstonia pickettii*	DQ997838	93
OTU-104	-	-	3.6	1.4	-	-	-	-	-	-	-	-	-	-	-	-	*Ralstonia pickettii*	NR_043152	100
OTU-105	-	-	-	-	-	-	-	-	-	0.6	-	-	-	-	-	-	*Cupriavidus pauculus*	AM418462	99
OTU-106	-	-	-	-	-	-	-	-	0.6	-	-	-	-	0.7	-	-	*Achromobacter xylosoxidans*	AB695331	99
OTU-107	-	-	-	-	-	-	2.0	-	-	-	-	-	0.6	1.4	-	-	*Achromobacter spanius*	KX527629	100
OTU-108	-	-	-	-	-	-	-	0.6	-	-	-	-	-	-	-	-	*Candidimonas nitroreducens*	MF480397	99
OTU-109	-	-	-	-	-	-	-	-	-	0.6	0.7	0.7	-	-	-	-	*Georgfuchsia toluolica*	NR_115995	94
OTU-110	-	-	-	-	-	-	-	-	-	0.6	-	-	-	-	-	-	*Georgfuchsia toluolica*	NR_115995	94
OTU-111	-	-	-	-	-	-	-	-	-	-	0.7	-	-	-	-	-	*Methylotenera mobilis*	NR_102842	97
OTU-112	-	-	-	-	-	-	-	-	-	-	-	0.7	-	-	-	-	*Noviherbaspirillum psychrotolerans*	NR_109468	98
OTU-113	-	-	-	-	-	-	0.7	-	-	-	-	-	-	-	-	-	*Janthinobacterium lividum*	MF979551	98
OTU-114	-	-	-	-	-	-	0.7	0.6	1.7	1.1	0.7	2.0	-	-	-	-	*Herminiimonas fonticola*	AB512142	100
OTU-115	1.1	-	0.6	-	-	-	-	-	-	-	1.5	-	-	-	-	-	*Duganella zoogloeoides*	LN812952	100
OTU-116	-	-	-	-	-	-	2.0	0.6	3.9	0.6	-	-	-	-	-	-	*Collimonas fungivorans*	AB740926	100
OTU-117	-	-	-	-	-	-	-	-	-	-	0.7	-	-	-	-	-	*Herbaspirillum lusitanum*	NR_028859	99
OTU-118	-	-	-	-	-	-	-	-	-	-	-	-	-	-	0.6	-	*Herbaspirillum hiltneri*	KU305712	96
OTU-119	-	-	-	0.7	-	-	-	-	-	1.7	-	-	-	-	-	-	*Undibacterium pigrum*	KP196819	98
OTU-120	-	-	-	-	-	-	-	-	-	0.6	-	-	-	-	-	-	*Undibacterium terreum*	NR_109599	98
OTU-121	-	-	-	-	-	-	-	-	-	0.6	1.5	1.3	-	-	-	-	*Janthinobacterium agaricidamnosum*	AY167838	99
OTU-122	-	-	-	-	-	-	-	-	-	0.6	-	-	-	-	-	-	*Duganella nigrescens*	EF584756	98
OTU-123	-	-	-	-	-	-	-	-	-	0.6	-	-	-	-	-	-	*Massilia aerilata*	HF585365	99
OTU-124	0.6	-	-	-	-	-	-	-	-	-	-	-	-	-	-	-	*Herbaspirillum huttiense*	KY124206	91
OTU-125	-	-	-	-	-	-	-	0.6	-	-	-	-	-	-	-	-	*Collimonas fungivorans*	AB740926	92
OTU-126	-	-	-	-	-	-	-	-	-	-	-	1.3	-	-	-	-	*Tepidimonas ignav*	NR_025041	90
OTU-127	-	-	-	-	-	-	0.7	-	-	-	-	-	2.9	3.4	0.6	0.6	*Stenotrophomonas rhizophila*	KX349202	100
OTU-128	-	-	-	-	-	-	-	-	-	-	-	0.7	-	-	0.6	-	*Pseudoxanthomonas yeongjuensis*	AB682413	99
OTU-129	-	-	-	-	-	-	-	-	0.6	-	-	-	-	-	-	-	*Stenotrophomonas chelatiphaga*	KJ561110	100
OTU-130	0.6	0.6	1.8	1.4	3.9	1.7	-	0.6	-	-	-	-	-	2.0	0.6	2.4	*Stenotrophomonas maltophilia*	CP015612	100
OTU-131	-	-	-	-	-	-	-	-	-	-	-	-	-	0.7	-	-	*Stenotrophomonas rhizophila*	KR065721	99
OTU-132	-	-	-	-	-	-	-	-	-	1.1	3.0	2.0	-	-	-	-	*Dokdonella soli*	NR_044554	99
OTU-133	-	-	-	-	-	-	-	-	-	-	0.7	-	-	-	-	-	*Dokdonella fugitiva*	NR_042397	97
OTU-134	-	-	-	-	-	-	-	-	-	0.6	-	-	-	-	-	-	*Dokdonella immobilis*	NR_108377	99
OTU-135	-	-	-	-	-	-	-	-	-	-	-	0.7	-	-	-	-	*Lysobacter korlensis*	NR_108236	97

Table S3. Continued

Libraries / Collections	Clone libraries (%)												Isolate collections (%)				Closest relatives in known species	Acc. No.	Identity (%)
Tissues	Leaf blade			Petiole			Tap root			Lateral root			Lateral root						
Experimental fields	NPK	PK	K	NPK	PK	K	NPK	PK	K	NPK	PK	K	PK		K				
Isolation medium	-	-	-	-	-	-	-	-	-	-	-	-	R2A	TSA	R2A	TSA			
Library / Collection name	LB-NPK	LB-PK	LB-K	PE-NPK	PE-PK	PE-K	TR-NPK	TR-PK	TR-K	LR-NPK	LR-PK	LR-K	LR-PK-R	LR-PK-T	LR-K-R	LR-K-T			
OTU-136	-	-	-	-	-	-	0.7	-	-	0.6	1.5	-	-	-	-	-	*Lysobacter ginsengisoli*	NR_112563	99
OTU-137	-	-	-	-	-	-	-	-	-	0.6	1.5	-	-	-	-	-	*Lysobacter niastensis*	JQ342848	100
OTU-138	-	-	-	-	-	-	0.7	-	-	-	-	-	-	-	-	-	*Lysobacter mobilis*	NR_134760	98
OTU-139	-	-	-	-	-	-	-	-	-	-	0.7	-	-	-	-	-	*Lysobacter pocheonensis*	EU273938	100
OTU-140	-	-	-	-	-	-	-	-	-	0.6	-	-	-	-	-	-	*Rhodanobacter humi*	KX674374	100
OTU-141	-	-	-	-	-	-	-	-	-	-	4.4	-	2.9	-	2.2	-	*Rhodanobacter soli*	NR_116741	99
OTU-142	-	-	-	-	-	-	-	-	0.6	0.6	-	-	0.6	-	-	-	*Rhodanobacter umsongensis*	NR_108435	99
OTU-143	-	-	-	-	-	-	-	-	-	-	-	-	0.6	-	-	-	*Rhodanobacter spathiphylli*	NR_042434	93
OTU-144	-	-	0.6	-	0.6	-	-	-	-	-	-	-	-	-	-	-	*Luteibacter rhizovicinu*	MF525359	99
OTU-145	-	-	-	-	-	-	-	-	-	0.6	-	-	-	-	-	-	*Rudaea cellulosilytica*	NR_044566	99
OTU-146	-	-	-	-	-	-	-	-	-	-	2.2	-	-	-	-	-	*Metallibacterium scheffleri*	KC257407	93
OTU-147	-	1.7	-	-	-	-	-	-	-	-	-	-	-	-	-	-	*Commensalibacter intestini*	LT631761	99
OTU-148	-	-	-	0.7	-	1.7	-	-	-	-	-	-	-	-	-	-	*Roseomonas mucosa*	MF525216	100
OTU-149	-	-	-	-	-	-	8.6	9.1	1.7	1.1	-	0.7	0.6	-	2.8	3.0	*Rhizobium herbae*	KX881443	99
OTU-150	-	-	-	-	-	-	-	-	-	-	-	-	-	1.4	-	-	*Rhizobium herbae*	KX881443	98
OTU-151	-	-	-	-	-	-	-	-	-	-	-	-	-	-	-	0.6	*Rhizobium giardinii*	KR014110	94
OTU-152	1.1	-	1.2	2.1	-	0.6	-	-	0.6	-	0.7	-	1.8	2.7	-	1.2	*Rhizobium metallidurans*	KX664481	100
OTU-153	-	-	-	-	-	-	-	-	1.1	-	-	-	-	-	-	-	*Rhizobium tubonense*	NR_116255	100
OTU-154	-	-	-	-	-	0.6	4.6	0.6	-	0.6	1.5	1.3	0.6	-	1.7	-	*Rhizobium grahamii*	KF477161	99
OTU-155	-	-	-	-	-	-	-	-	1.1	-	0.7	0.7	4.7	-	2.2	-	*Rhizobium alamii*	KU305699	100
OTU-156	1.1	-	-	-	0.6	-	0.7	-	-	-	-	-	-	-	-	-	*Ochrobactrum pseudogrignonense*	JX393010	99
OTU-157	-	-	-	-	0.6	-	-	-	-	-	-	-	-	-	-	-	*Aureimonas rubiginis*	NR_118424	98
OTU-158	-	-	-	-	0.6	-	-	-	-	-	-	-	-	-	-	-	*Aureimonas rubiginis*	NR_118424	93
OTU-159	-	-	-	-	-	0.6	-	-	-	-	-	-	-	-	-	-	*Aureimonas ureilytica*	NR_043995	99
OTU-160	-	-	-	0.7	-	-	-	-	-	-	-	-	-	-	-	-	*Aureimonas pseudogalii*	NR_151994	99
OTU-161	-	-	-	-	0.6	-	-	-	-	-	-	-	-	-	-	-	*Rhizobium qilianshanense*	NR_132606	99
OTU-162	-	-	-	-	-	-	-	1.2	-	-	-	-	-	-	0.6	0.6	*Tianweitania sediminis*	NR_148593	99
OTU-163	0.0	0.6	0.6	0.7	0.6	-	2.0	1.8	1.1	-	-	-	-	2.7	-	0.6	*Agrobacterium tumefaciens*	KF673154	100
OTU-164	1.1	2.8	3.0	2.8	2.2	5.6	0.7	3.0	1.7	-	-	-	0.6	2.7	2.2	8.4	*Rhizobium nepotum*	MG576194	100
OTU-165	-	-	-	-	-	-	-	0.6	-	-	-	-	-	-	-	-	*Neorhizobium alkalisoli*	LN774523	98
OTU-166	-	-	-	-	-	-	-	1.2	1.7	2.2	3.7	2.0	2.9	-	7.8	-	*Neorhizobium galegae*	KF356027	100
OTU-167	-	-	-	-	-	-	-	-	0.6	-	-	-	-	0.7	-	-	*Rhizobium daejeonense*	HF585487	98
OTU-168	-	-	0.6	-	-	-	-	-	-	-	-	-	-	-	1.1	-	*Ensifer mexicanus*	MG016487	98
OTU-169	-	-	-	-	-	-	-	-	-	0.6	-	-	-	-	0.6	-	*Mesorhizobium gobiense*	KJ556378	98
OTU-170	-	-	-	-	-	-	-	-	-	-	-	-	4.1	3.4	1.1	-	*Aminobacter anthyllidis*	HG974525	98
OTU-171	-	-	-	-	-	-	2.6	5.5	6.7	-	-	2.0	6.4	-	5.6	-	*Mesorhizobium loti*	AP017605	100
OTU-172	5.1	2.8	0.6	13.8	10.1	9.6	-	1.2	0.6	-	-	-	-	-	-	-	*Phyllobacterium myrsinacearum*	NR_113874	100
OTU-173	-	-	-	-	-	-	-	-	-	-	-	-	0.6	1.4	-	0.6	*Phyllobacterium myrsinacearum*	AY512821	99
OTU-174	-	-	-	-	-	-	-	-	-	-	-	-	-	1.4	-	-	*Phyllobacterium ifriqiyense*	MG461619	100
OTU-175	0.6	-	-	-	-	-	-	-	-	-	-	-	-	-	-	-	*Bartonella elizabethae*	AB246807	98
OTU-176	-	-	-	-	-	-	0.7	-	-	-	-	-	-	-	0.6	0.6	*Aminobacter aminovorans*	KJ689311	100
OTU-177	-	-	-	-	0.6	-	-	-	-	-	-	-	-	-	-	-	*Rhizobium rosettiformans*	KJ401121	97
OTU-178	-	-	-	-	-	-	-	-	-	1.1	0.7	0.7	-	-	1.1	-	*Devosia insulae*	NR_044036	99
OTU-179	-	-	-	-	-	-	1.3	1.2	3.4	-	0.7	-	3.5	6.8	3.9	7.8	*Devosia neptuniae*	MF796810	100
OTU-180	-	-	-	-	-	-	-	-	-	-	-	-	0.6	-	-	-	*Devosia lucknowensis*	NR_132697	99

Table S3. Continued

Libraries / Collections	Clone libraries (%)												Isolate collections (%)				Closest relatives in known species	Acc. No.	Identity (%)
Tissues	Leaf blade			Petiole			Tap root			Lateral root			Lateral root						
Experimental fields	NPK	PK	K	NPK	PK	K	NPK	PK	K	NPK	PK	K	PK		K				
Isolation medium	-	-	-	-	-	-	-	-	-	-	-	-	R2A	TSA	R2A	TSA			
Library / Collection name	LB-NPK	LB-PK	LB-K	PE-NPK	PE-PK	PE-K	TR-NPK	TR-PK	TR-K	LR-NPK	LR-PK	LR-K	LR-PK-R	LR-PK-T	LR-K-R	LR-K-T			
OTU-181	-	-	-	-	-	-	-	-	-	-	-	-	-	1.4	-	-	*Devosia riboflavina*	AF501346	99
OTU-182	-	-	-	-	-	-	-	-	-	-	-	1.3	-	-	-	-	*Devosia ginsengisoli*	KC676321	97
OTU-183	-	-	-	-	-	-	-	-	1.1	-	-	-	-	-	-	-	*Paradevosia shaoguanensis*	NR_136441	97
OTU-184	-	-	-	-	-	-	-	-	-	-	-	0.7	-	-	-	-	*Novosphingobium arabidopsis*	NR_133799	99
OTU-185	-	-	-	-	-	-	-	-	-	0.6	-	-	-	-	-	-	*Novosphingobium hassiacum*	NR_028962	98
OTU-186	-	-	-	-	-	-	-	1.2	0.6	0.6	-	-	-	-	2.2	-	*Novosphingobium barchaimii*	NR_118314	98
OTU-187	-	-	-	-	0.6	-	15.8	22.0	15.2	2.2	8.1	5.3	1.8	2.0	8.3	1.2	*Novosphingobium naphthalenivorans*	AB649005	99
OTU-188	-	-	-	-	-	-	-	-	-	-	-	0.7	-	-	-	-	*Sphingosinicella vermicomposti*	KU133480	98
OTU-189	-	-	-	-	-	-	0.7	-	2.8	-	1.5	2.0	0.6	-	3.3	-	*Sphingobium aromaticiconvertens*	NR_042479	98
OTU-190	-	-	-	-	-	-	-	-	-	-	-	-	-	-	0.6	-	*Sphingobium czechense*	KR088386	99
OTU-191	-	-	-	-	-	-	-	-	-	0.6	1.5	1.3	12.9	-	5.0	-	*Sphingomonas asaccharolytica*	NR_113761	100
OTU-192	1.1	0.6	0.6	4.1	1.7	1.1	-	-	-	-	-	-	0.6	-	-	-	*Sphingomonas melonis*	HE716911	99
OTU-193	0.6	-	1.2	-	-	-	-	-	-	-	-	-	-	-	-	-	*Sphingomonas faeni*	MG561802	100
OTU-194	-	-	-	-	-	-	-	-	-	-	-	-	-	-	0.6	-	*Sphingomonas desiccabilis*	NR_042372	98
OTU-195	0.6	0.6	-	-	-	-	-	-	-	-	-	-	-	-	-	-	*Sphingomonas yunnanensis*	EU730917	98
OTU-196	1.7	1.7	-	7.6	7.3	3.4	-	-	-	-	-	-	-	-	-	-	*Sphingomonas zeae*	NR_136793	100
OTU-197	-	-	-	-	-	-	-	-	-	-	-	-	-	-	2.2	-	*Sphingomonas ginsengisoli*	KX504221	99
OTU-198	-	-	-	-	-	-	-	-	-	-	-	-	-	-	0.6	-	*Altererythrobacter epoxidivorans*	KC921173	99
OTU-199	-	-	-	-	-	-	-	-	-	-	-	-	3.5	0.7	2.2	-	*Sphingopyxis taejonensis*	AB795553	100
OTU-200	-	-	-	-	-	-	-	-	-	-	-	0.7	-	-	-	-	*Nordella oligomobilis*	NR_114615	97
OTU-201	-	-	-	-	-	-	-	-	-	-	0.7	-	-	-	-	-	*Nordella oligomobilis*	KU258275	94
OTU-202	-	-	-	-	-	-	-	-	-	0.6	-	-	-	-	-	-	*Hyphomicrobium facile*	Y14312	100
OTU-203	-	-	-	0.7	-	-	-	-	-	-	-	-	-	-	-	-	*Hyphomicrobium sulfonivorans*	NR_025082	96
OTU-204	-	-	-	-	-	-	-	-	-	-	-	0.7	-	-	-	-	*Labrys wisconsinensis*	NR_116004	96
OTU-205	-	-	-	-	-	-	-	-	-	0.6	-	-	-	-	-	-	*Labrys wisconsinensis*	NR_116004	94
OTU-206	-	-	-	-	-	-	-	0.6	-	-	-	-	-	-	-	-	*Kaistia geumhonensis*	NR_108141	99
OTU-207	-	-	-	-	-	0.6	-	-	-	-	-	-	-	-	-	-	*Methylocapsa palsarum*	NR_137418	98
OTU-208	0.6	0.6	-	-	-	-	-	-	-	-	-	-	-	-	-	-	*Methylosinus sporium*	KF981557	93
OTU-209	-	0.6	-	-	-	-	-	-	-	-	-	-	-	-	-	-	*Methylobacterium cerastii*	NR_117118	99
OTU-210	2.9	13.0	8.4	13.8	14.0	29.8	-	-	-	-	-	-	-	-	-	-	*Methylobacterium goesingense*	MG778820	99
OTU-211	12.6	14.1	6.0	2.1	6.7	13.5	-	-	-	-	-	-	-	-	-	-	*Methylobacterium fujisawaense*	KT720195	100
OTU-212	-	1.1	-	4.8	9.0	1.1	-	-	0.6	-	-	-	-	-	-	-	*Methylobacterium populi*	MG778712	100
OTU-213	-	1.1	-	3.4	2.8	1.1	-	-	-	-	-	-	-	-	-	-	*Methylobacterium aquaticum*	KR920749	99
OTU-214	-	-	-	-	-	-	-	0.6	0.6	-	-	-	0.6	1.4	0.6	3.0	*Bosea lupini*	KF730777	100
OTU-215	-	-	-	-	-	-	-	-	-	-	0.7	-	-	-	-	-	*Bradyrhizobium elkanii*	EU573787	98
OTU-216	-	-	0.6	-	0.6	-	2.6	2.4	7.3	1.1	1.5	1.3	0.6	-	-	-	*Bradyrhizobium embrapense*	NR_145861	100
OTU-217	-	-	-	-	-	-	2.0	3.0	3.4	-	-	-	-	-	-	-	*Rhodopseudomonas faecalis*	LC066638	99
OTU-218	-	-	-	-	-	-	2.6	1.8	4.5	0.6	0.7	-	0.6	-	1.7	-	*Tardiphaga robiniae*	KX881440	100
OTU-219	-	-	-	-	-	0.6	-	-	-	-	-	-	-	-	-	-	*Rhodoplanes oryzae*	LC178576	95
OTU-220	-	-	-	-	-	-	-	1.2	1.1	-	-	-	-	-	-	-	*Pseudolabrys taiwanensis*	EU938323	95
OTU-221	-	-	-	-	-	-	-	0.6	0.6	1.1	-	-	-	-	-	-	*Phenylobacterium immobile*	NR_026498	98
OTU-222	-	-	-	-	-	-	-	-	-	-	0.7	-	-	-	-	-	*Phenylobacterium aquaticus*	KT309087	98
OTU-223	-	-	-	-	-	-	-	-	-	1.1	0.7	1.3	1.2	-	0.6	-	*Caulobacter henricii*	AM921622	99
OTU-224	-	-	-	-	-	-	-	-	-	-	-	-	-	0.7	-	-	*Brevundimonas bullata*	MG576189	100
OTU-225	-	-	-	-	-	-	0.7	-	-	-	-	-	1.2	0.7	-	-	*Brevundimonas vesicularis*	MG819328	100

33

Table S3. Continued

Libraries / Collections	Clone libraries (%)												Isolate collections (%)				Closest relatives in known species	Acc. No.	Identity (%)
Tissues	Leaf blade			Petiole			Tap root			Lateral root			Lateral root						
Experimental fields	NPK	PK	K	NPK	PK	K	NPK	PK	K	NPK	PK	K	PK		K				
Isolation medium	-	-	-	-	-	-	-	-	-	-	-	-	R2A	TSA	R2A	TSA			
Library / Collection name	LB-NPK	LB-PK	LB-K	PE-NPK	PE-PK	PE-K	TR-NPK	TR-PK	TR-K	LR-NPK	LR-PK	LR-K	LR-PK-R	LR-PK-T	LR-K-R	LR-K-T			
OTU-226	-	-	-	-	-	-	-	-	-	-	0.7	-	2.3	-	0.6	-	*Asticcacaulis benevestitus*	NR_042433	99
OTU-227	-	-	-	-	-	-	-	-	-	0.6	-	-	-	-	-	-	*Rhizomicrobium electricum*	NR_108115	85
OTU-228	-	-	-	-	-	-	0.7	-	-	-	-	-	-	-	-	-	*Rhodoplanes piscinae*	LC178578	89
OTU-229	-	-	-	-	-	-	-	-	-	0.6	-	0.7	-	-	-	-	*Rhizomicrobium electricum*	NR_108115	92
OTU-230	-	-	-	-	-	-	-	-	-	-	0.7	-	-	-	-	-	*Pleomorphomonas oryzae*	NR_114056	90
OTU-231	-	-	-	-	-	-	-	-	0.6	-	-	-	-	-	1.7	-	*Inquilinus ginsengisoli*	NR_112560	100
OTU-232	-	-	-	-	-	-	-	0.6	-	-	-	-	-	-	-	-	*Lacibacterium aquatile*	NR_125556	91
OTU-233	-	-	-	-	-	-	0.7	-	-	-	-	-	-	-	-	-	*Ca.* Paracaedibacter acanthamoebae	CP008941	95
OTU-234	-	-	-	-	-	-	-	-	0.6	-	-	-	-	-	-	-	*Reyranella soli*	NR_109674	99
OTU-235	-	-	-	-	-	-	-	-	0.6	-	-	-	-	-	-	-	*Enhydrobacter aerosaccus*	NR_113385	97
OTU-236	-	-	-	-	-	-	-	-	0.6	-	-	-	-	-	-	-	*Anaeromyxobacter dehalogenans*	KC921178	87
OTU-237	-	-	-	-	-	-	-	0.6	-	-	-	-	-	-	-	-	*Anaeromyxobacter dehalogenans*	KF952438	87
OTU-238	-	-	-	-	-	-	-	-	-	1.7	-	0.7	-	-	-	-	*Chondromyces pediculatus*	GU207875	91
OTU-239	-	-	-	-	-	-	-	-	-	-	0.7	-	-	-	-	-	*Jahnella thaxteri*	NR_117461	91
OTU-240	-	-	-	-	-	-	-	-	-	0.6	-	-	-	-	-	-	*Byssovorax cruenta*	NR_042341	97
OTU-241	-	-	-	-	-	-	-	-	-	-	-	0.7	-	-	-	-	*Kofleria flava*	HF937255	87
OTU-242	-	-	-	-	-	-	-	-	-	0.6	-	-	-	-	-	-	*Kofleria flava*	HF543825	88
OTU-243	-	-	-	-	-	-	-	-	-	-	-	0.7	-	-	-	-	*Jahnella thaxteri*	NR_117461	85
OTU-244	-	-	-	-	-	-	-	-	-	-	0.7	-	-	-	-	-	*Sandaracinus amylolyticus*	KP306728	90
OTU-245	-	-	-	-	-	-	-	0.6	-	-	-	-	-	-	-	-	*Pyxidicoccus fallax*	MF163327	95
OTU-246	-	-	-	-	-	-	-	-	-	-	-	0.7	-	-	-	-	*Geobacter metallireducens*	NR_075011	82
OTU-247	-	-	-	-	-	-	-	-	0.6	-	-	-	-	-	-	-	*Desulfuromonas acetexigens*	NR_044770	82
OTU-248	-	-	-	-	-	-	-	0.6	0.6	-	-	-	-	-	-	-	*Geobacter metallireducens*	NR_075011	82
OTU-249	-	-	-	-	-	-	-	-	-	0.6	-	-	-	-	-	-	*Bdellovibrio bacteriovorus*	KU973532	97
OTU-250	-	-	-	-	-	-	-	-	-	0.6	-	-	-	-	-	-	*Bdellovibrio bacteriovorus*	CP002190	89
OTU-251	-	-	-	-	-	-	-	-	0.6	-	-	-	-	-	-	-	*Bdellovibrio bacteriovorus*	AJ278145	87
OTU-252	-	-	-	-	-	-	-	-	-	-	-	-	0.6	-	-	-	*Agreia pratensis*	KX036606	100
OTU-253	0.6	-	-	-	-	-	0.7	-	-	-	-	-	-	-	-	-	*Frondihabitans peucedani*	NR_116933	99
OTU-254	-	-	-	0.7	-	-	-	-	-	-	-	-	-	-	-	-	*Frigoribacterium faeni*	LN774274	99
OTU-255	-	2.8	-	-	-	-	-	-	-	-	-	-	-	-	-	-	*Curtobacterium herbarum*	MG778849	100
OTU-256	-	-	-	-	-	-	-	-	-	-	-	-	0.6	0.7	-	-	*Okibacterium fritillariae*	KR476456	99
OTU-257	-	-	0.6	-	-	-	-	-	-	-	-	-	0.6	-	-	-	*Leifsonia kafniensis*	KJ589435	99
OTU-258	-	-	-	-	-	-	-	-	-	-	-	-	-	-	-	0.6	*Microbacterium shaanxiense*	MF801349	99
OTU-259	-	-	-	-	-	-	-	-	-	-	-	-	-	1.4	-	-	*Microbacterium murale*	NR_117603	100
OTU-260	-	-	-	-	-	-	-	-	-	-	-	-	5.3	13.5	0.6	0.6	*Microbacterium phyllosphaerae*	MF796703	100
OTU-261	-	-	-	-	-	-	-	-	-	-	-	-	-	1.4	-	-	*Microbacterium esteraromaticum*	EU036992	100
OTU-262	0.6	-	-	-	-	-	-	-	-	-	-	-	-	0.7	0.6	0.6	*Microbacterium testaceum*	KY027168	100
OTU-263	-	-	-	-	-	-	-	0.6	-	-	-	-	-	-	-	-	*Microbacterium ginsengisoli*	KC342051	98
OTU-264	-	-	-	-	-	-	-	-	0.6	-	-	-	1.2	0.7	-	0.6	*Leifsonia xyli*	HQ530514	99
OTU-265	2.9	2.8	4.2	4.8	0.6	2.2	-	-	-	-	-	-	0.6	-	0.6	3.0	*Paenarthrobacter nicotinovorans*	MF796793	100
OTU-266	-	-	-	-	-	-	-	-	-	-	-	-	0.6	0.7	0.6	-	*Micrococcus aloeverae*	KX418589	100
OTU-267	0.6	-	-	-	-	-	-	-	-	-	-	-	-	-	-	-	*Rothia amarae*	KU605695	100
OTU-268	-	-	-	-	-	-	-	0.6	-	-	-	-	-	-	-	-	*Arsenicicoccus bolidensis*	NR_025598	100
OTU-269	-	-	-	-	-	-	-	0.6	-	-	-	-	-	-	-	-	*Actinomadura cremea*	JX860397	100
OTU-270	-	-	-	-	-	-	-	-	-	-	-	-	-	-	0.6	-	*Streptomyces rishiriensis*	KU647228	96

Table S3. Continued

Libraries / Collections	Clone libraries (%)												Isolate collections (%)				Closest relatives in known species	Acc. No.	Identity (%)
Tissues	Leaf blade			Petiole			Tap root			Lateral root			Lateral root						
Experimental fields	NPK	PK	K	NPK	PK	K	NPK	PK	K	NPK	PK	K	PK		K				
Isolation medium	-	-	-	-	-	-	-	-	-	-	-	-	R2A	TSA	R2A	TSA			
Library / Collection name	LB-NPK	LB-PK	LB-K	PE-NPK	PE-PK	PE-K	TR-NPK	TR-PK	TR-K	LR-NPK	LR-PK	LR-K	LR-PK-R	LR-PK-T	LR-K-R	LR-K-T			
OTU-271	-	-	-	-	-	-	2.6	2.4	1.1	3.3	-	4.0	12.3	18.9	9.4	23.5	*Streptomyces rishiriensis*	KU647228	100
OTU-272	-	-	-	-	-	-	-	-	-	1.7	0.7	0.7	2.3	3.4	-	-	*Streptomyces olivochromogenes*	KJ573037	100
OTU-273	-	-	-	-	-	-	-	-	-	-	-	-	-	2.0	0.6	1.2	*Streptomyces flavogriseus*	KX358692	100
OTU-274	-	-	-	-	-	-	-	-	-	-	-	-	0.6	1.4	-	-	*Kitasatospora aureofaciens*	MF526714	99
OTU-275	-	-	-	-	-	-	-	-	-	-	-	-	-	-	-	0.6	*Streptomyces thermocarboxydus*	KT163798	93
OTU-276	-	-	-	-	-	-	-	-	-	-	-	-	1.2	-	-	7.8	*Streptomyces griseoaurantiacus*	KY412831	100
OTU-277	-	-	-	-	-	-	-	-	-	-	-	-	-	-	-	0.6	*Streptomyces xiamenensis*	KX358649	100
OTU-278	-	-	-	-	-	-	-	-	-	-	-	-	1.8	2.0	-	-	*Streptomyces aomiensis*	NR_112998	100
OTU-279	-	-	-	-	-	-	-	-	-	-	-	-	-	0.7	-	-	*Streptomyces scabrisporus*	FJ486372	100
OTU-280	-	-	-	-	-	-	-	-	-	-	-	-	-	0.7	-	-	*Streptomyces aomiensis*	NR_112998	94
OTU-281	-	-	-	-	-	-	-	-	-	0.6	-	-	-	-	-	-	*Kutzneria chonburiensis*	NR_145619	100
OTU-282	-	-	-	-	-	-	-	-	-	0.6	-	-	-	-	-	-	*Actinoplanes octamycinicus strain*	NR_112131	98
OTU-283	-	-	-	-	-	-	0.7	-	-	-	-	-	-	-	-	-	*Cellulomonas denverensis*	AB491158	99
OTU-284	-	-	-	-	-	-	-	-	-	0.6	-	-	-	-	-	-	*Catenulispora graminis*	NR_109018	99
OTU-285	-	-	-	-	-	-	-	-	-	-	-	-	0.6	-	-	-	*Nakamurella panacisegetis*	KJ606832	95
OTU-286	-	-	-	-	-	-	1.3	-	-	-	-	-	-	-	-	0.6	*Mycobacterium frederiksbergense*	HG941823	99
OTU-287	0.6	-	0.6	-	-	-	-	0.6	-	-	-	-	-	-	-	-	*Mycobacterium hodleri*	FN796799	100
OTU-288	-	-	-	-	-	-	-	-	-	-	-	-	0.6	-	-	-	*Mycobacterium goodii*	DQ447773	99
OTU-289	-	-	-	-	-	-	0.7	-	-	-	-	-	-	-	-	-	*Mycobacterium smegmatis*	JN049510	98
OTU-290	-	-	-	-	-	-	0.7	0.6	-	-	-	-	-	-	-	-	*Mycobacterium moriokaense*	KC494311	99
OTU-291	-	-	-	-	-	-	-	-	0.6	-	-	-	-	-	-	-	*Mycobacterium moriokaense*	KC494311	97
OTU-292	-	-	-	-	-	-	-	-	0.6	-	-	-	-	-	-	-	*Mycobacterium flavescens*	KX954376	96
OTU-293	-	-	-	2.8	0.6	-	-	0.6	0.6	-	-	-	0.6	-	-	0.6	*Mycobacterium abscessus*	AP018436	100
OTU-294	-	-	-	-	-	-	-	-	-	-	-	-	0.6	0.7	-	0.6	*Rhodococcus jostii*	MF796707	99
OTU-295	-	-	-	-	-	-	0.7	-	0.6	-	-	-	-	0.7	-	0.6	*Rhodococcus erythropolis*	MG722792	100
OTU-296	-	0.6	-	-	-	-	-	-	-	-	-	-	-	0.7	-	-	*Rhodococcus fascians*	MG205633	100
OTU-297	-	-	-	-	-	-	-	-	-	-	-	-	-	0.7	-	-	*Nocardia tengchongensis*	NR_152644	100
OTU-298	-	-	-	0.7	-	-	-	-	-	-	-	-	-	-	-	-	*Lawsonella clevelandensis*	NR_151867	99
OTU-299	-	0.6	-	-	-	-	-	-	-	-	-	-	-	-	-	-	*Corynebacterium efficiens*	BA000035	99
OTU-300	-	-	-	-	-	-	-	-	-	-	-	-	-	-	0.6	-	*Nocardioides albus*	KC800806	100
OTU-301	-	-	-	-	-	-	-	-	-	-	-	-	2.3	-	-	-	*Nocardioides ganghwensis*	JF505976	100
OTU-302	-	-	-	-	-	-	-	-	-	-	-	-	1.8	-	0.6	-	*Nocardioides hankookensis*	JX841081	99
OTU-303	-	-	-	-	-	0.6	-	-	-	-	-	-	-	-	-	-	*Nocardioides islandensis*	NR_044235	94
OTU-304	-	1.1	-	-	-	-	-	-	-	-	-	-	-	-	-	-	*Marmoricola aquaticus*	JN615437	98
OTU-305	-	-	-	-	-	-	-	-	-	-	-	-	-	0.7	-	-	*Aeromicrobium kwangyangensis*	EU834249	99
OTU-306	-	-	-	-	-	-	-	-	-	-	-	-	-	-	0.6	-	*Glycomyces lechevalierae*	NR_025790	100
OTU-307	-	-	-	0.7	-	-	-	-	-	-	-	-	-	-	-	-	*Cutibacterium acnes*	LC341280	100
OTU-308	-	-	-	-	-	-	-	-	-	-	-	-	0.6	0.7	-	0.6	*Curtobacterium flaccumfaciens*	MG819350	97
OTU-309	-	-	-	-	0.6	-	-	-	-	-	-	-	-	-	-	-	*Conexibacter woesei*	NR_074830	90
OTU-310	-	-	-	-	0.6	-	-	-	-	-	-	-	-	-	-	-	*Solirubrobacter phytolaccae*	NR_133858	92
OTU-311	-	-	-	-	-	-	-	-	-	-	0.7	-	-	-	-	-	*Gaiella occulta*	NR_118138	94
OTU-312	-	-	-	-	-	-	-	-	-	-	-	-	-	0.7	-	-	*Bacillus subtilis*	KY680229	100
OTU-313	0.6	-	-	-	-	1.1	-	-	-	-	-	-	-	-	-	0.6	*Bacillus pumilus*	LC010662	99
OTU-314	0.6	-	-	-	-	-	-	-	-	-	-	-	-	0.7	-	-	*Bacillus thuringiensis*	MG651420	99
OTU-315	-	-	-	-	-	-	-	-	-	-	-	0.7	-	-	-	-	*Bacillus benzoevorans*	NR_115578	98

Table S3. Continued

Libraries / Collections	Clone libraries (%)												Isolate collections (%)				Closest relatives in known species	Acc. No.	Identity (%)
Tissues	Leaf blade			Petiole			Tap root			Lateral root			Lateral root						
Experimental fields	NPK	PK	K	NPK	PK	K	NPK	PK	K	NPK	PK	K	PK		K				
Isolation medium	-	-	-	-	-	-	-	-	-	-	-	-	R2A	TSA	R2A	TSA			
Library / Collection name	LB-NPK	LB-PK	LB-K	PE-NPK	PE-PK	PE-K	TR-NPK	TR-PK	TR-K	LR-NPK	LR-PK	LR-K	LR-PK-R	LR-PK-T	LR-K-R	LR-K-T			
OTU-316	-	-	-	-	-	-	-	-	-	-	-	-	-	0.7	0.6	1.2	*Bacillus megaterium*	MG819312	100
OTU-317	-	-	-	2.1	0.6	-	-	-	-	-	-	-	-	-	-	-	*Staphylococcus epidermidis*	MG815839	99
OTU-318	-	-	-	0.7	-	-	-	-	-	-	-	-	-	-	-	-	*Staphylococcus epidermidis*	MG815839	97
OTU-319	-	-	-	1.4	-	-	-	-	-	-	-	-	-	-	-	-	*Staphylococcus hominis*	CP014107	99
OTU-320	0.6	-	-	-	-	-	-	-	-	-	-	-	-	-	-	-	*Enterococcus plantarum*	NR_118050	96
OTU-321	0.6	0.6	-	-	-	-	-	-	-	-	-	-	-	-	-	-	*Enterococcus termitis*	NR_042406	99
OTU-322	-	-	0.6	-	-	-	-	-	-	-	-	-	-	-	-	-	*Enterococcus mundtii*	MG031213	99
OTU-323	-	1.7	-	-	0.6	-	-	-	-	-	-	-	-	-	-	-	*Enterococcus sulfureus*	KR265371	100
OTU-324	0.6	-	-	-	-	-	-	-	-	-	-	-	-	-	-	-	*Carnobacterium maltaromaticum*	MG807447	100
OTU-325	-	-	-	0.7	-	-	-	-	-	-	-	-	-	-	-	-	*Carnobacterium divergens*	KJ958216	99
OTU-326	-	-	-	-	-	-	-	-	-	-	-	-	-	-	-	0.6	*Solibacillus isronensis*	KY038743	99
OTU-327	-	-	-	-	-	-	-	-	-	-	-	-	-	0.7	-	-	*Lysinibacillus fusiformis*	GQ501071	97
OTU-328	-	-	-	-	-	-	0.7	-	-	-	-	-	-	-	-	-	*Sporosarcina psychrophila*	NR_113752	99
OTU-329	1.7	-	-	2.8	-	-	1.3	-	0.6	-	-	-	-	0.7	-	1.2	*Bacillus gibsonii*	MG651558	100
OTU-330	0.6	-	-	-	-	-	-	-	-	-	-	-	-	-	-	-	*Bacillus xiaoxiensis*	KY849468	99
OTU-331	-	-	0.6	-	-	-	-	-	-	-	-	-	-	-	-	-	*Lactococcus lactis*	MG815845	99
OTU-332	0.6	-	-	-	-	-	-	-	-	-	-	-	-	-	-	-	*Lactococcus garvieae*	MF155911	100
OTU-333	-	-	-	-	1.1	-	-	-	-	-	-	-	-	-	-	-	*Streptococcus gordonii*	GU561385	100
OTU-334	1.1	-	-	-	-	-	-	-	0.6	-	-	-	-	-	-	-	*Paenibacillus amylolyticus*	MF524306	100
OTU-335	-	0.6	-	-	-	-	-	-	-	-	-	-	-	-	-	-	*Paenibacillus rigui*	KY660606	97
OTU-336	-	-	-	-	-	-	0.7	-	-	-	-	-	-	-	-	-	*Paenibacillus wynnii*	MG561907	98
OTU-337	-	-	-	-	-	-	-	0.6	-	-	-	-	-	-	-	-	*Paenibacillus periandrae*	NR_148839	92
OTU-338	-	-	-	0.7	-	-	-	-	-	-	-	-	-	-	-	-	*Paenibacillus daejeonensis*	AY032949	99
OTU-339	-	-	-	-	-	-	-	-	-	-	-	0.7	-	-	-	-	*Anaerotaenia torta*	AB298768	94
OTU-340	-	1.1	-	-	-	-	-	-	-	-	-	-	-	-	-	-	*Clostridium bolteae*	NR_113410	95
OTU-341	-	-	-	-	-	-	-	-	-	0.6	-	-	-	-	-	-	*Clostridium disporicum*	NR_026491	99
OTU-342	-	-	-	-	-	-	-	-	-	-	-	0.7	-	-	-	-	*Ca.* Koribacte	CP000360	92
OTU-343	-	-	-	-	-	-	-	-	-	-	-	0.7	-	-	-	-	*Ca.* Koribacter	CP000360	92
OTU-344	-	-	-	-	-	-	-	-	-	-	0.7	-	-	-	-	-	*Ca*. Koribacter versatilis	CP000360	94
OTU-345	-	-	-	-	-	-	-	-	-	-	-	0.7	-	-	-	-	*Terriglobus roseus*	KJ529056	96
OTU-346	-	-	-	-	-	-	-	-	-	0.6	0.7	-	-	-	-	-	*Edaphobacter modestus*	NR_115813	98
OTU-347	-	-	-	-	-	-	-	-	-	-	-	0.7	-	-	-	-	*Aridibacter famidurans*	NR_133697	96
OTU-348	-	-	-	-	-	-	-	-	-	1.1	-	-	-	-	-	-	*Stenotrophobacter terrae*	NR_146023	94
OTU-349	-	-	-	-	-	-	-	-	-	-	-	1.3	-	-	-	-	*Paludibaculum fermentans*	NR_134120	90
OTU-350	-	-	-	-	-	-	-	-	-	0.6	-	-	-	-	-	-	*Paludibaculum fermentans*	NR_134120	91
OTU-351	-	-	-	-	-	-	-	-	-	0.6	-	-	-	-	-	-	*Vicinamibacter silvestris*	KP761690	90
OTU-352	-	-	-	-	-	-	-	-	-	-	0.7	-	-	-	-	-	*Vicinamibacter silvestris*	KP761690	89
OTU-353	-	-	-	-	-	-	-	-	-	1.1	-	-	-	-	-	-	*Geothrix fermentans*	HF559176	94
OTU-354	-	-	-	-	-	-	-	-	-	5.6	1.5	5.3	-	-	-	-	*Niastella koreensis*	AB682408	90
OTU-355	-	-	-	-	-	-	-	-	-	0.6	0.7	-	-	-	-	-	*Niastella vici*	NR_148859	95
OTU-356	-	-	-	-	-	-	-	-	-	-	0.7	0.7	-	-	-	-	*Niastella koreensis*	AB682408	93
OTU-357	-	-	-	-	-	-	-	-	-	-	0.7	-	-	-	-	-	*Niastella vici*	NR_148859	95
OTU-358	-	-	-	-	-	-	-	-	-	-	-	2.0	-	-	-	-	*Niastella gongjuensis*	NR_137250	94
OTU-359	-	-	-	-	-	-	-	-	-	-	0.7	0.7	-	-	-	-	*Niastella populi*	NR_116486	94
OTU-360	-	-	-	-	-	-	-	-	-	-	0.7	-	-	-	-	-	*Niastella vici*	NR_148859	96

Table S3. Continued

Libraries / Collections	Clone libraries (%)												Isolate collections (%)				Closest relatives in known species	Acc. No.	Identity (%)
Tissues	Leaf blade			Petiole			Tap root			Lateral root			Lateral root						
Experimental fields	NPK	PK	K	NPK	PK	K	NPK	PK	K	NPK	PK	K	PK		K				
Isolation medium	-	-	-	-	-	-	-	-	-	-	-	-	R2A	TSA	R2A	TSA			
Library / Collection name	LB-NPK	LB-PK	LB-K	PE-NPK	PE-PK	PE-K	TR-NPK	TR-PK	TR-K	LR-NPK	LR-PK	LR-K	LR-PK-R	LR-PK-T	LR-K-R	LR-K-T			
OTU-361	-	-	-	-	-	-	-	-	-	1.1	3.7	0.7	-	-	-	-	*Niastella gongjuensis*	NR_137250	98
OTU-362	-	-	-	-	-	-	-	-	-	0.6	-	1.3	-	-	-	-	*Niastella populi*	NR_116486	98
OTU-363	-	-	-	-	-	-	-	-	-	3.9	2.2	-	-	-	-	-	*Niastella yeongjuensis*	AB682409	99
OTU-364	-	-	-	-	-	-	-	-	-	1.1	-	0.7	-	-	-	-	*Flavitalea populi*	NR_117796	96
OTU-365	-	-	-	-	-	-	-	-	-	2.2	2.2	0.7	-	-	-	-	*Flavitalea gansuensis*	NR_108737	94
OTU-366	-	-	-	-	-	-	-	-	-	-	-	0.7	-	-	-	-	*Ferruginibacter lapsinanis*	NR_044589	98
OTU-367	-	-	-	-	-	-	-	-	-	-	0.7	-	-	-	-	-	*Ferruginibacter profundus*	NR_148259	98
OTU-368	-	-	-	-	-	-	-	-	-	0.6	0.7	-	-	-	-	-	*Ferruginibacter profundus*	NR_148259	95
OTU-369	-	-	-	-	-	-	-	-	-	0.6	-	-	-	-	-	-	*Ferruginibacter paludis*	NR_136802	93
OTU-370	-	-	-	-	-	-	-	-	-	-	-	0.7	-	-	-	-	*Flavitalea gansuensis*	NR_108737	91
OTU-371	-	-	-	-	-	-	-	-	-	0.6	-	-	-	-	-	-	*Flavitalea populi*	NR_117796	93
OTU-372	-	-	-	-	-	-	-	-	-	0.6	-	-	-	-	-	-	*Flavitalea populi*	NR_117796	94
OTU-373	-	-	-	-	-	-	-	-	-	0.6	-	-	-	-	-	-	*Flavitalea populi*	NR_117796	94
OTU-374	-	-	-	-	-	-	-	-	-	-	-	0.7	-	-	-	-	*Niabella ginsengisoli*	NR_044541	93
OTU-375	-	-	-	-	-	-	-	-	-	-	0.7	-	-	-	-	-	*Terrimonas arctica*	NR_134213	93
OTU-376	-	-	-	-	-	-	-	-	-	-	0.7	0.7	-	-	-	-	*Niabella tibetensis*	NR_117487	94
OTU-377	-	-	-	-	-	-	-	-	-	1.7	-	-	-	-	-	-	*Flavitalea populi*	NR_117796	93
OTU-378	-	-	-	-	-	-	-	-	-	-	0.7	-	-	-	-	-	*Terrimonas lutea*	NR_041250	95
OTU-379	-	-	-	-	-	-	-	-	-	0.6	-	-	-	-	-	-	*Chitinophaga oryziterrae*	NR_109375	99
OTU-380	-	-	-	-	-	-	-	-	-	0.6	-	0.7	-	-	0.6	-	*Chitinophaga arvensicola*	JN819560	99
OTU-381	-	-	-	-	-	-	-	-	-	0.6	-	-	-	-	-	-	*Chitinophaga rupis*	NR_116917	99
OTU-382	-	-	-	-	-	-	-	-	-	0.6	-	-	-	-	-	-	*Portibacter lacus*	NR_113569	86
OTU-383	-	-	-	-	-	-	-	-	-	-	-	0.7	0.6	-	-	-	*Mucilaginibacter rigui*	KY302294	97
OTU-384	-	-	-	-	-	-	-	-	-	-	-	-	0.6	-	-	-	*Mucilaginibacter rigui*	NR_113976	100
OTU-385	-	-	-	-	-	-	-	-	-	1.7	-	-	-	-	-	-	*Mucilaginibacter dorajii*	NR_117444	99
OTU-386	-	-	-	-	-	-	-	-	-	-	-	0.7	-	-	-	-	*Mucilaginibacter dorajii*	NR_117444	96
OTU-387	-	-	-	-	-	-	-	-	-	-	0.7	-	-	-	-	-	*Mucilaginibacter calamicampi*	NR_118563	99
OTU-388	-	-	-	-	-	-	-	-	-	-	-	-	-	-	0.6	-	*Pedobacter boryungensis*	JX971550	98
OTU-389	-	-	-	-	-	-	-	-	-	-	-	0.7	-	-	-	-	*Solitalea koreensis*	NR_044568	88
OTU-390	-	-	-	-	-	-	-	-	-	-	-	0.7	-	-	-	-	*Mucilaginibacter gynuensis*	NR_118549	86
OTU-391	-	-	-	-	-	-	-	-	-	-	-	0.7	-	-	-	-	*Flavobacterium piscis*	HE774303	99
OTU-392	-	-	-	-	-	-	-	-	-	-	-	-	-	-	2.2	-	*Flavobacterium chilense*	KT369933	98
OTU-393	-	-	-	-	-	-	-	-	-	-	-	-	-	-	0.6	-	*Chryseobacterium xinjiangense*	NR_131771	95
OTU-394	-	-	-	-	-	-	-	-	-	-	0.7	-	-	-	-	-	*Chryseolinea serpens*	NR_108511	91
OTU-395	-	-	-	-	-	-	-	-	-	-	0.7	-	-	-	-	-	*Chryseolinea serpens*	NR_108511	89
OTU-396	-	-	-	-	-	-	-	-	-	1.7	-	-	-	-	-	-	*Chryseolinea serpens*	NR_108511	99
OTU-397	-	-	-	-	-	-	-	-	-	0.6	-	-	-	-	-	-	*Fulvivirga kasyanovii*	AB433335	89
OTU-398	-	-	-	-	-	-	-	-	-	0.6	-	0.7	-	-	-	-	*Dyadobacter fermentans*	KU350606	100
OTU-399	-	-	-	-	-	-	1.3	0.6	1.1	-	-	-	-	-	-	-	*Chthoniobacter flavus*	NR_115225	97
OTU-400	-	-	-	-	-	-	1.3	0.6	-	-	-	0.7	-	-	-	-	*Chthoniobacter flavus*	NR_115225	97
OTU-401	-	-	-	-	-	-	0.7	-	-	-	-	-	-	-	-	-	*Chthoniobacter flavus*	NR_115225	91
OTU-402	-	-	-	-	-	-	0.7	-	-	-	-	-	-	-	-	-	*Ca.* Xiphinematobacter brevicolli	AF217462	90
OTU-403	-	-	-	-	-	-	-	0.6	-	-	-	-	-	-	-	-	*Ca.* Xiphinematobacter brevicolli	AF217462	90
OTU-404	-	-	-	-	-	-	-	-	1.1	-	-	-	-	-	-	-	*Prosthecobacter dejongeii*	NR_026021	98
OTU-405	-	-	-	-	-	-	-	-	-	-	-	0.7	-	-	-	-	*Luteolibacter cuticulihirudinis*	NR_109603	95

Table S3. Continued

Libraries / Collections	Clone libraries (%)												Isolate collections (%)				Closest relatives in known species	Acc. No.	Identity (%)
Tissues	Leaf blade			Petiole			Tap root			Lateral root			Lateral root						
Experimental fields	NPK	PK	K	NPK	PK	K	NPK	PK	K	NPK	PK	K	PK		K				
Isolation medium	-	-	-	-	-	-	-	-	-	-	-	-	R2A	TSA	R2A	TSA			
Library / Collection name	LB-NPK	LB-PK	LB-K	PE-NPK	PE-PK	PE-K	TR-NPK	TR-PK	TR-K	LR-NPK	LR-PK	LR-K	LR-PK-R	LR-PK-T	LR-K-R	LR-K-T			
OTU-406	-	-	-	-	-	-	0.7	0.6	-	0.6	0.7	1.3	-	-	-	-	*Luteolibacter arcticus*	NR_135889	99
OTU-407	-	-	-	-	-	-	-	-	-	-	-	0.7	-	-	-	-	*Lacunisphaera limnophila*	NR_146376	95
OTU-408	-	-	-	-	-	-	-	-	-	-	0.7	-	-	-	-	-	*Lacunisphaera limnophila*	NR_146376	97
OTU-409	-	-	-	-	-	-	-	-	-	-	-	0.7	-	-	-	-	*Lacunisphaera anatis*	NR_146377	95
OTU-410	-	-	-	-	-	-	-	-	-	1.1	-	-	-	-	-	-	*Opitutus terrae*	KF228164	95
OTU-411	-	-	0.6	-	-	-	-	-	-	-	-	-	-	-	-	-	*Ca.* Babela massiliensis	HG793133	90
OTU-412	-	-	-	-	-	0.6	-	-	-	-	-	-	-	-	-	-	*Ca.* Babela massiliensis	HG793133	91
OTU-413	-	-	-	-	-	-	-	-	0.6	-	-	-	-	-	-	-	*Ca.* Babela massiliensis	HG793133	91
OTU-414	-	-	-	-	-	-	0.7	-	-	-	-	-	-	-	-	-	*Ca.* Babela massiliensis	HG793133	90
OTU-415	-	-	-	-	-	-	-	0.6	-	-	-	-	-	-	-	-	*Ca.* Babela massiliensis	HG793133	86
OTU-416	-	-	-	-	-	-	-	-	2.2	-	-	-	-	-	-	-	*Ca.* Babela massiliensis	HG793133	83
OTU-417	-	-	-	-	-	-	-	-	0.6	0.6	-	-	-	-	-	-	*Schlesneria paludicola*	NR_042466	99
OTU-418	-	-	-	-	-	-	-	0.6	-	0.6	-	-	-	-	-	-	*Schlesneria paludicola*	NR_042466	94
OTU-419	-	-	-	-	-	-	0.7	-	0.6	1.1	-	-	-	-	-	-	*Planctopirus limnophila*	NR_074670	91
OTU-420	-	-	-	-	-	-	-	-	0.6	-	-	-	-	-	-	-	*Planctopirus limnophila*	NR_074670	88
OTU-421	-	0.6	-	-	-	-	-	-	1.1	-	-	-	-	-	-	-	*Planctopirus limnophila*	NR_074670	88
OTU-422	-	-	-	-	-	-	-	-	0.6	-	-	-	-	-	-	-	*Rubinisphaera brasiliensis*	JX307094	86
OTU-423	-	-	-	-	-	-	0.7	-	-	-	-	-	-	-	-	-	*Gimesia maris*	KF228168	90
OTU-424	-	-	-	-	-	-	-	0.6	-	-	-	-	-	-	-	-	*Gimesia maris*	KF228168	93
OTU-425	-	-	-	-	-	-	-	-	0.6	-	-	-	-	-	-	-	*Gimesia maris*	KF228168	90
OTU-426	-	-	-	-	-	-	0.7	1.2	-	-	-	-	-	-	-	-	*Pirellula staleyi*	NR_074521	87
OTU-427	-	-	-	-	-	-	-	0.6	-	-	-	-	-	-	-	-	*Pirellula staleyi*	NR_074521	89
OTU-428	-	-	-	-	-	-	-	-	-	0.6	-	-	-	-	-	-	*Pirellula staleyi*	NR_074521	87
OTU-429	-	-	-	-	-	-	-	-	0.6	-	-	-	-	-	-	-	*Ca.* Anammoximicrobium moscowii	KC467065	85
OTU-430	-	-	-	-	-	-	-	-	-	-	-	0.7	-	-	-	-	*Bythopirellula goksoyri*	NR_118636	88
OTU-431	-	-	0.6	-	-	-	0.7	-	0.6	-	-	-	-	-	-	-	*Gemmata massiliana*	NR_148576	99
OTU-432	-	-	-	-	-	-	-	-	0.6	-	-	-	-	-	-	-	*Gemmata massiliana*	NR_148576	93
OTU-433	-	-	-	-	-	-	0.7	-	-	-	-	-	-	-	-	-	*Gemmata massiliana*	NR_148576	89
OTU-434	-	-	-	-	-	-	-	0.6	-	-	-	-	-	-	-	-	*Gemmata obscuriglobus*	X85248	90
OTU-435	-	-	-	-	-	-	0.7	-	-	-	-	-	-	-	-	-	*Zavarzinella formosa*	NR_042465	98
OTU-436	-	-	-	-	-	-	-	-	-	-	0.7	-	-	-	-	-	*Gemmata massiliana*	NR_148576	90
OTU-437	-	-	-	-	-	-	0.7	0.6	1.1	-	-	0.7	-	-	-	-	*Singulisphaera acidiphila*	NR_102439	98
OTU-438	-	-	-	-	-	-	-	-	-	-	0.7	-	-	-	-	-			
OTU-439	-	-	-	-	-	-	-	-	-	-	-	0.7	-	-	-	-	*Fimbriimonas ginsengiso*	CP007139	88
OTU-440	-	-	-	-	-	-	-	-	-	-	1.5	-	-	-	-	-	*Fimbriimonas ginsengisoli*	NR_121726	88
OTU-441	-	-	-	-	-	-	-	-	-	0.6	-	-	-	-	-	-	*Fimbriimonas ginsengisoli*	CP007139	91
OTU-442	-	-	-	-	-	-	-	-	-	0.6	-	-	-	-	-	-	*Deinococcus aquiradiocola*	NR_041487	99
OTU-443	-	-	-	-	-	0.6	-	-	-	-	-	-	-	-	-	-	*Deinococcus geothermalis*	CP000359	100
OTU-444	-	-	-	-	-	-	-	-	-	-	-	0.7	-	-	-	-	*Gemmatirosa kalamazoonesis*	CP007128	85
OTU-445	-	-	-	-	-	-	-	-	-	-	0.7	-	-	-	-	-	*Gemmatimonas aurantiaca*	KF228166	89
OTU-446	-	-	-	-	-	-	-	-	-	-	0.7	-	-	-	-	-	*Gemmatimonas aurantiaca*	NR_074708	89
OTU-447	-	-	-	-	-	0.6	-	-	-	-	-	-	-	-	-	-	*Dehalogenimonas alkenigignens*	NR_109657	88
OTU-448	-	-	-	0.7	-	-	-	-	-	-	-	-	-	-	-	-			
OTU-449	-	-	-	-	-	-	0.7	-	-	-	-	-	-	-	-	-	*Solemya pervernicosa gill symbiont*	AB499617	85
OTU-450	-	-	-	-	-	-	-	-	-	-	-	0.7	-	-	-	-	*Thermomarinilinea lacunifontana*	NR_132293	82

38

Table S3. Continued

Libraries / Collections	Clone libraries (%)												Isolate collections (%)				Closest relatives in known species	Acc. No.	Identity (%)
Tissues	Leaf blade			Petiole			Tap root			Lateral root			Lateral root						
Experimental fields	NPK	PK	K	NPK	PK	K	NPK	PK	K	NPK	PK	K	PK		K				
Isolation medium	-	-	-	-	-	-	-	-	-	-	-	-	R2A	TSA	R2A	TSA			
Library / Collection name	LB-NPK	LB-PK	LB-K	PE-NPK	PE-PK	PE-K	TR-NPK	TR-PK	TR-K	LR-NPK	LR-PK	LR-K	LR-PK-R	LR-PK-T	LR-K-R	LR-K-T			
OTU-451	-	-	-	-	-	-	-	-	-	0.6	-	-	-	-	-	-	*Ornatilinea apprim*	NR_109544	81
OTU-452	-	-	-	-	-	-	-	-	-	-	-	0.7	-	-	-	-	*Thermomarinilinea lacunifontana*	NR_132293	83
OTU-453	-	-	-	-	-	-	-	-	-	-	0.7	-	-	-	-	-	*Ornatilinea apprima*	NR_109544	83
OTU-454	-	-	-	-	-	-	-	-	-	-	0.7	0.7	-	-	-	-	*Thermomarinilinea lacunifontana*	NR_132293	83
OTU-455	-	-	-	-	-	-	-	-	-	0.6	-	-	-	-	-	-	*Thermanaerothrix daxensis*	NR_117865	84
OTU-456	-	-	-	-	-	-	-	-	-	-	-	0.7	-	-	-	-	*Kouleothrix aurantiaca*	AB079640	90

OTUs (Operational taxonomic units) were defined at 97% sequence identity by using MOTHUR.

Table S4. List of bacterial isolates used for the inoculation test on sugar beet seedlings

OTU No.	Isolates	BlastN search results with partial sequence of 16S rRNA genes[a]	
		Closest knwon species	Identity (%)
	Actinobacteria		
OTU-264	*Leifsonia* sp. TK069 (LC040755)	*Leifsonia xyli* (HQ530514)	99
OTU-260	*Microbacterium* sp. TK080 (LC040765)	*Microbacterium phyllosphaerae* (MF796703)	100
OTU-302	*Nocardioides* sp. RP084 (LC040865)	*Nocardioides hankookensis* (JX841081)	99
OTU-301	*Nocardioides* sp. RP110 (LC040866)	*Nocardioides cavernae* (NR_156135)	100
OTU-271	*Streptomyces* sp. RP008 (LC040224)	*Streptomyces humidus* (KU324480)	100
OTU-278	*Streptomyces* sp. RP073 (LC040284)	*Streptomyces aomiensis* (NR_112998)	100
OTU-276	*Streptomyces* sp. TK192 (LC040867)	*Streptomyces griseoaurantiacus* (KY412831)	100
OTU-272	*Streptomyces* sp. TP071 (LC040603)	*Streptomyces olivochromogenes* (NR_112483)	100
	Bacilli		
OTU-316	*Bacillus* sp. TK156 (LC040868)	*Bacillus aryabhattai* (KY777355)	100
OTU-329	*Bacillus* sp. TP182 (LC040689)	*Bacillus gibsonii* (MG651558)	100
	Alphaproteobacteria		
OTU-226	*Asticcacaulis* sp. RK043 (LC040869)	*Asticcacaulis benevestitus* (NR_042433)	99
OTU-214	*Bosea* sp. TK063 (LC040870)	*Bosea robiniae* (NR_108516)	98
OTU-216	*Bradyrhizobium* sp. RP196 (LC040385)	*Bradyrhizobium japonicum* (KY000642)	100
OTU-223	*Caulobacter* sp. RK066 (LC040442)	*Caulobacter henricii* (MG982457)	99
OTU-178	*Devosia* sp. No.184 (LC040871)	*Devosia insulae* (NR_044036)	99
OTU-179	*Devosia* sp. TK103 (LC040784)	*Devosia neptuniae* (MF796810)	100
OTU-171	*Mesorhizobium* sp. RK190 (LC040872)	*Mesorhizobium gobiense* (KJ556378)	100
OTU-171	*Mesorhizobium* sp. RP126 (LC040326)	*Mesorhizobium huakuii* (KP331546)	99
OTU-170	*Mesorhizobium* sp. TP027 (LC040873)	*Mesorhizobium chacoense* (NR_025411)	98
OTU-166	*Neorhizobium* sp. RK064 (LC602160)	*Neorhizobium galegae* (HG938355)	100
OTU-186	*Novosphingobium* sp. RK010 (LC040874)	*Novosphingobium barchaimii* (NR_118314)	98
OTU-187	*Novosphingobium* sp. RK036 (LC040415)	*Novosphingobium naphthalenivorans* (AB649005)	99
OTU-187	*Novosphingobium* sp. RK163 (LC040527)	*Novosphingobium lindaniclasticum* (MG516209)	99
OTU-187	*Novosphingobium* sp. RK193 (LC040875)	*Novosphingobium resinovorum* (KU305726)	99
OTU-173	*Phyllobacterium* sp. TP192 (LC040876)	*Phyllobacterium myrsinacearum* (AY512821)	99
OTU-154	*Rhizobium* sp. RK005 (LC040877)	*Rhizobium grahamii* (JX841042)	100
OTU-155	*Rhizobium* sp. RK061 (LC040438)	*Rhizobium gallicum* (AY509211)	100
OTU-149	*Rhizobium* sp. TK032 (LC040728)	*Rhizobium giardinii* (HQ263115)	99
OTU-152	*Rhizobium* sp. TP011 (LC040563)	*Rhizobium etli* (KX008304)	100
OTU-189	*Sphingobium* sp. RK166 (LC040530)	*Sphingobium aromaticiconvertens* (MF101093)	98
OTU-197	*Sphingomonas* sp. RK102 (LC040472)	*Sphingomonas ginsengisoli* (KX504221)	98
OTU-199	*Sphingomonas* sp. RK106 (LC040475)	*Sphingomonas taejonensis* (NR_024999)	100
OTU-191	*Sphingomonas* sp. RP089 (LC040298)	*Sphingomonas kwangyangensis* (EF693741)	98
OTU-191	*Sphingomonas* sp. RP195 (LC040384)	*Sphingomonas asaccharolytica* (NR_113761)	100
OTU-218	*Tardiphaga* sp. RK140 (LC602161)	*Tardiphaga robiniae* (CP050292)	99
	Betaproteobacteria		
OTU-87	*Polaromonas* sp. RK103 (LC040879)	*Polaromonas ginsengisoli* (AB245355)	100
OTU-86	*Variovorax* sp. RK170 (LC040880)	*Variovorax paradoxus* (CP002417)	100
	Gammaproteobacteria		
OTU-69	*Pantoea* sp. RK126 (LC040494)	*Pantoea ananatis* (KX011138)	96
OTU-74	*Pantoea* sp. TK182 (LC040852)	*Pantoea ananatis* (KX011138)	98
OTU-37	*Pseudomonas* sp. TP131 (LC040646)	*Pseudomonas fluorescens* (KU351091)	100
OTU-37	*Pseudomonas* sp. TP180 (LC040881)	*Pseudomonas fluorescens* (KP418807)	99
OTU-96	*Rhizobacter* sp. RK021 (LC040878)	*Methylibium petroleiphilum* (CP000555)	99
OTU-141	*Rhodanobacter* sp. RK114 (LC040483)	*Rhodanobacter spathiphylli* (KY078839)	100
OTU-141	*Rhodanobacter* sp. RP069 (LC040280)	*Rhodanobacter soli* (NR_116741)	99

[a]Results with approximately 500 bp are shown.
Gray highlight indicates the isolates described in main text.

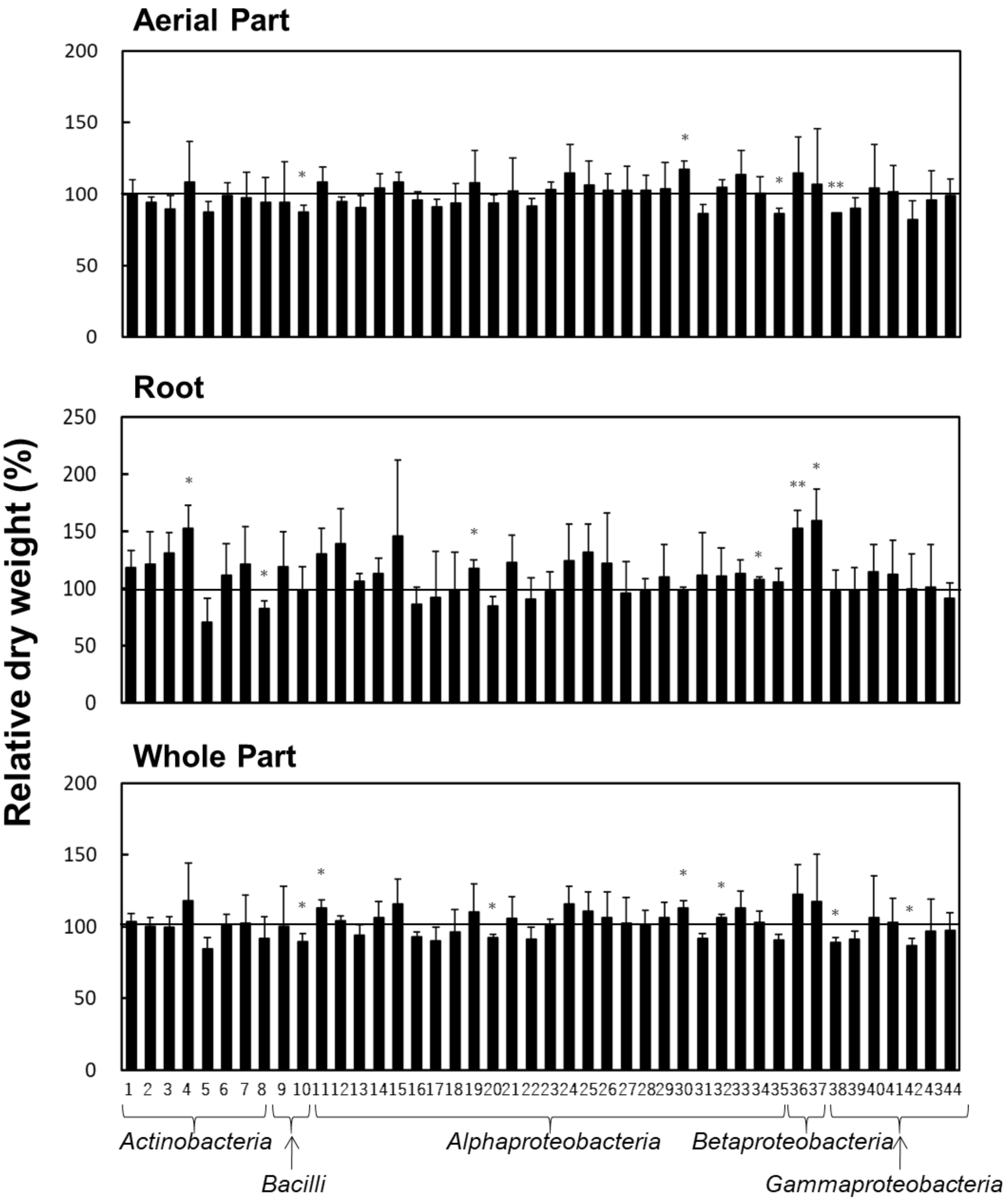

Fig. S1. Screening of sugar beet growth-promoting bacteria. Forty-four lateral root-associated bacterial isolates were inoculated onto sugar beet seedlings. After 4 weeks of cultivation, dry weights were measured. Control plants were inoculated with sterilized water. Twelve seedlings in a tray were used in an inoculation test with each isolate, and this test was repeated three or four times at different dates for ensuring the reproducibility of PGP effects. The dry weights of inoculated seedlings were compared to those of control seedlings (non-inoculated seedlings) by Welch's *t*-test (two-tailed). Single and double asterisks indicate statistical significance at $P < 0.05$ and $P < 0.01$, respectively. Bacterial isolates: 4, *Nocardioides* sp. RP110; 8, *Streptomyces* sp. TP071; 10, *Bacillus* sp. TP182; 11, *Asticcacaulis* sp. RK043; 19, *Mesorhizobium* sp. TP027; 20, *Neorhizobium* sp. RK064; 30, *Sphingobium* sp. RK166; 32, *Sphingopyxis* sp. RK106; 34, *Sphingomonas* sp. RP195; 35, *Tardiphaga* sp. RK140; 36, *Polaromonas* sp. RK103; 37, *Variovorax* sp. RK170; 38, *Pantoea* sp. RK126; and 42, *Rhizobacter* sp. RK021.

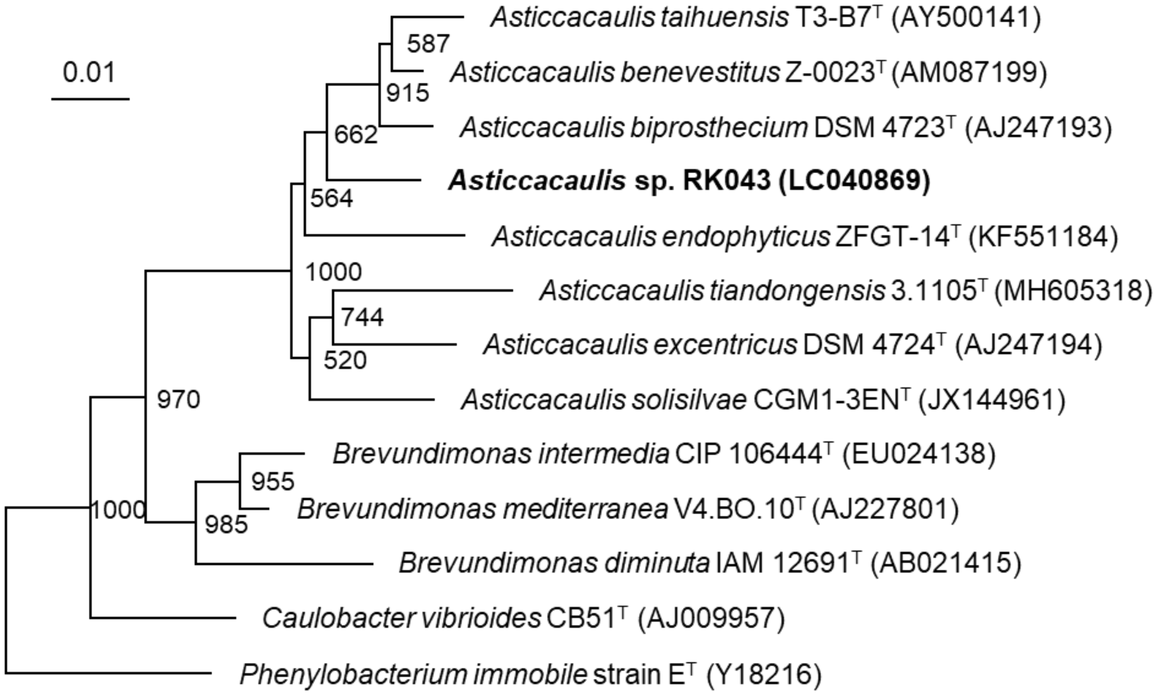

Fig. S2. Phylogenic tree analysis of the 16S rRNA gene sequence of *Asticcacaulis* sp. RK043. The tree was constructed using the neighbor-joining method with the sequences of a closely related type strain. The accession numbers are given in parentheses. The sequence of *Phenylobacterium immobile* was used as an outgroup. The numbers at the nodes are the proportions of 1000 bootstrap replicates, and values exceeding 500 are shown.

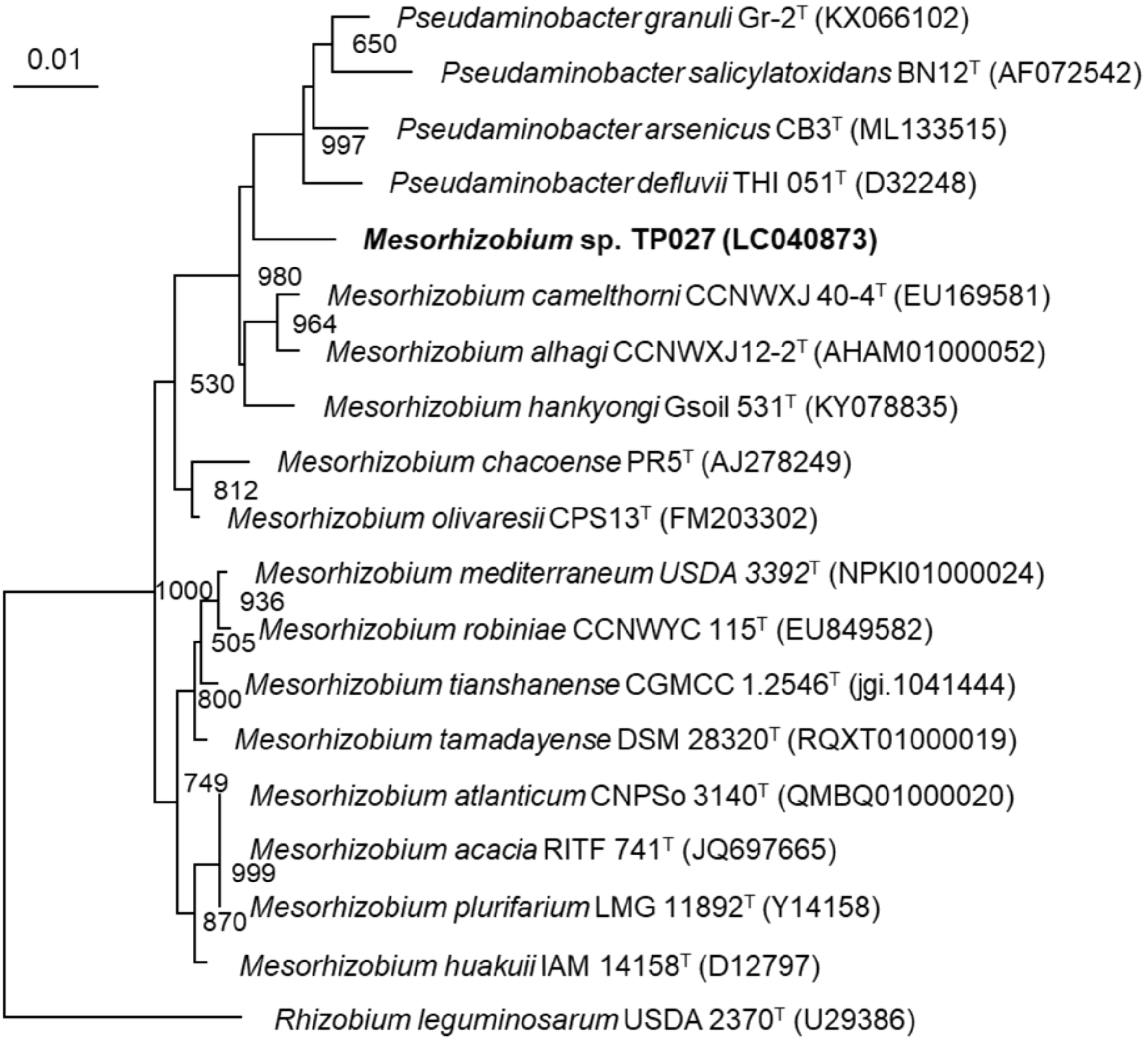

Fig. S3. Phylogenic tree analysis of the 16S rRNA gene sequences of *Mesorhizobium* sp. TP027. The tree was constructed using the neighbor-joining method with the sequences of a closely related type strain. The accession numbers are given in parentheses. The sequence of *Rhizobium leguminosarum* was used as an outgroup. The numbers at the nodes are the proportions of 1000 bootstrap replicates, and values exceeding 500 are shown.

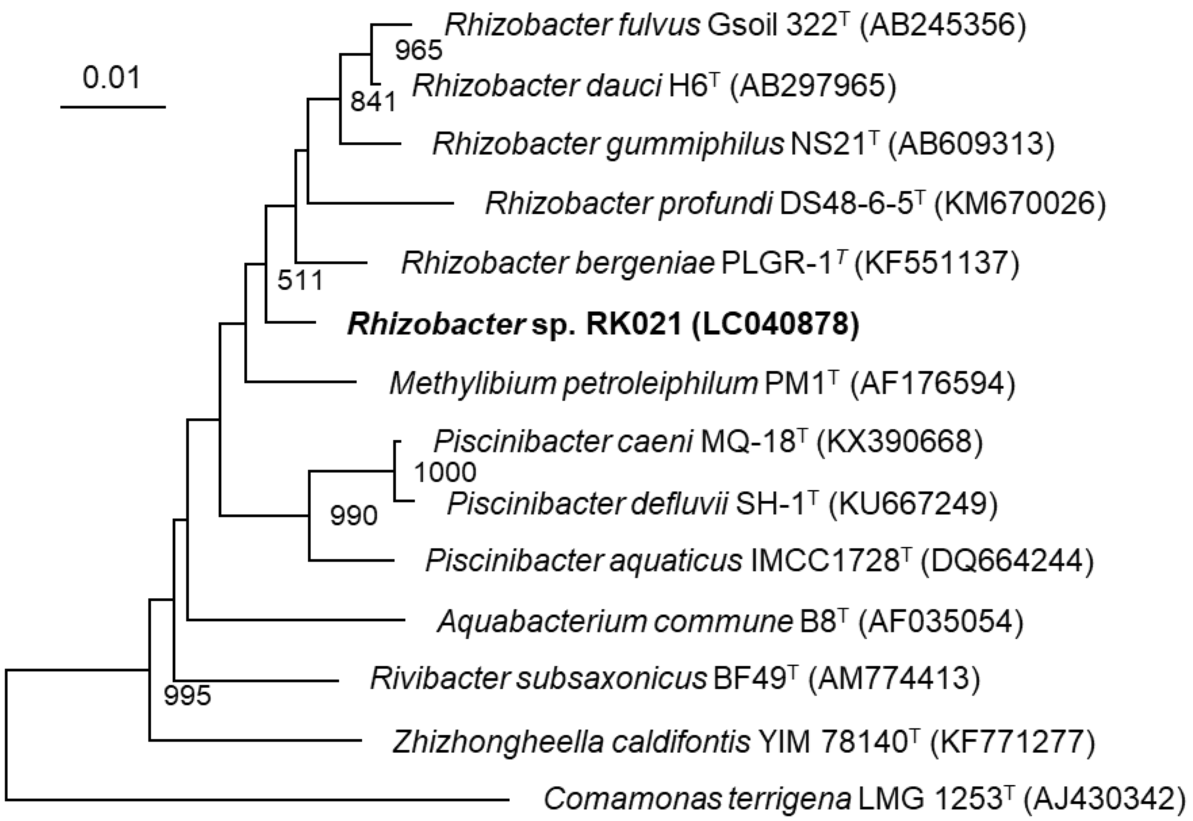

Fig. S4. Phylogenic tree analysis of the 16S rRNA gene sequence of *Rhizobacter* sp. RK021. The tree was constructed using the neighbor-joining method with the sequences of a closely related type strain. The isolates in the present study are shown in bold font. The accession numbers are given in parentheses. The sequence of *Comamonas terrigena* was used as an outgroup. The numbers at the nodes are the proportions of 1000 bootstrap replicates, and values exceeding 500 are shown
